# Supplementary material for: Design, Synthesis, Docking, DFT, MD Simulation Studies of a New Nicotinamide-Based Derivative: In Vitro Anticancer and VEGFR-2 Inhibitory Effects
Source: Molecules. 2022 Jul 19;27(14):4606. doi: 10.3390/molecules27144606 (PMC9317904; doi:10.3390/molecules27144606)
Supplement: Supplementary file 1 [file molecules-27-04606-s001.zip › molecules-1822310-supplementary.pdf]

Design, synthesis, docking, DFT, MD simulation studies of a new nicotinamide derivative: in vitro anticancer and VEGFR-2 inhibitory effects

Eslam B. Elkaeed [1], Reda G. Yousef [2], Hazem Elkady [2], Ibraheem M. M. Gobaara [3], Bshra A. Alsfouk [4], Dalal Z. Husein [5], Ibrahim M. Ibrahim [6], Ahmed M. Metwaly [7,8,*] and Ibrahim H. Eissa [2,*]

[1] Department of Pharmaceutical Sciences, College of Pharmacy, AlMaarefa University, Riyadh 13713,
Saudi Arabia; ikaeed@mcst.edu.sa

[2] Pharmaceutical Medicinal Chemistry & Drug Design Department, Faculty of Pharmacy (Boys), Al-Azhar University, Cairo11884, Egypt; redayousof@azhar.edu.eg (R.G.Y.); hazemelkady@azhar.edu.eg (H.E.)

[3] Zoology Department, Faculty of Science (Boys), Al-Azhar University, Cairo11884, Egypt; ibraheemgobaara@azhar.edu.eg

[4] Department of Pharmaceutical Sciences, College of Pharmacy, Princess Nourah bint Abdulrahman
University, P.O. Box 84428, Riyadh 11671, Saudi Arabia; baalsfouk@pnu.edu.sa

[5] Chemistry Department, Faculty of Science, New Valley University, El-Kharja 72511, Egypt; dalal_husein@sci.nvu.edu.eg

[6] Biophysics Department, Faculty of Science, Cairo University, Cairo 12613, Egypt; iabdelmagid@sci.cu.edu.eg

[7] Pharmacognosy and Medicinal Plants Department, Faculty of Pharmacy (Boys), Al-Azhar University,
Cairo 11884, Egypt

[8] Biopharmaceutical Products Research Department, Genetic Engineering and Biotechnology Research
Institute, City of Scientific Research and Technological Applications (SRTA-City), Alexandria 21934, Egypt

* Correspondence: ametwaly@azhar.edu.eg (A.M.M.); ibrahimeissa@azhar.edu.eg (I.H.E.)

Content

1	**Biological testing**<br>✓ Safety assay<br>✓ In vitro anti-proliferative activity.<br>✓ Selectivity index (SI)<br>✓ In vitro VEGFR-2 kinase assay.
2	**In silico studies**<br>✓ Docking studies<br>✓ MD simulation<br>✓ MMPBSA<br>✓ DFT<br>✓ ADMET studies<br>✓ Toxicity studies
3	**Spectral data**
4	**Report of in silico toxicity studies**

Molecular Docking studies

Crystal structure of VEGFR-2 [PDB ID: PDB ID: 2OH4, resolution: 2.05 Å] was obtained from the Protein Data Bank. The docking investigation was accomplished using MOE2014 software. At first, the crystal structure of VEGFR-2 was prepared by removing water molecules. Only one chain was retained besides the co-crystallized ligand (sorafenib). Then, the selected chain was protonated and subjected to the minimization of the energy process. Next, the active site of the target protein was defined.

Structures of the synthesized compound and sorafenib were drawn using ChemBioDraw Ultra 14.0 and saved as MDL-SD format. Such a file was opened using MOE to display the 3D structures which were protonated and subjected to energy minimization. Formerly, validation of the docking process was performed by docking the co-crystallized ligand against the isolated pocket of the active site. The produced RMSD value indicated the validity of the process. Finally, docking of the tested compounds was done through the dock option inserted in the compute window. For each docked molecule, 30 docked poses were produced using ASE for scoring function and force field for refinement. The results of the docking process were then visualized using Discovery Studio 4.0 software.

Molecular Dynamic Simulation:

We used the CHARMM-GUI web server to create the appropriate files for molecular dynamic (MD) simulation of the protein-compound complex. We utilized the TIP3P water model with a padding of 10 to solvate the system in a cubic box before neutralizing it by adding Na^+ and Cl^- ions to the physiological concentration of 0.154 M. The system was parameterized using the CHAMM36m force field, while the ligand was parameterized using the CHARMM general force field (CGenFF) tool implemented in CHARMM-GUI. To run the simulation with periodic boundary conditions (PBC), GROMACS 2021 was used as an MD engine. First, using the steepest descent algorithm, a minimization step was performed to remove any atom clashes. When the maximal force was less than 100 $KJ.mol^{-1}.nm^{-1}$, the minimization was considered to be converged. Two equilibration steps were performed starting with a constant number of atoms, constant volume, and constant temperature (NVT) ensemble, followed by a constant number of atoms, constant pressure, and constant temperature (NPT) ensemble. The temperature was set to 310 K and was maintained by the V-rescale algorithm. While the pressure was set to 1 atmospheric pressure and was maintained through Berendsen barostat. Finally, a 100 ns production run in NVT ensemble was performed. The bond lengths of hydrogen-bonded atoms were constrained using the LINear Constraint Solver (LINCS) method in each of the previous stages. The electrostatics were calculated using the Particle Mesh Ewald (PME) technique with a threshold of 1.2 nm. The Newtonian equations of motion were integrated using the leap-frog algorithm with a time step of 1 femtosecond for the equilibration steps and 2 femtoseconds for the production step. Before analyzing the trajectory, the PBC was removed using GROMACS built-in tool trjconv. The production run was saved each 100 picoseconds with a total of 1000 frames for each system. The analysis of the production trajectory was performed using VMD TK scripts. Root mean square deviation (RMSD), root mean square fluctuation (RMSF), solvent accessible surface area (SASA), radius of gyration (RoG), and the number of hydrogen bonds were calculated. To get a representative frame for each cluster, the trajectory was clustered using TTClust. First, backbone alignment was performed before determining the optimum number of clusters

using the elbow method. For each representative frame, protein–ligand interaction profiler (PLIP) was used to detect the number and types of interactions.

MM-GBSA Studies

Gmx_MMPBSA package was used to calculate the binding free energy using Molecular Mechanics Generalized Born Surface Area (MM-GBSA) algorithm with decomposition analysis to get the binding energies of amino acids within 10 Å around the ligand. The salt concentration and the method of solvation (igb) were set to 0.154 M and 5, respectively. The internal and external dielectric constant were set to 1.0 and 80.0, respectively, and other options were set as default. MM-GBSA approach is depicted in Equation 1

$\Delta G = < Gcomplex - Greceptor - Gligand >$ (1)

Where < > represents the average of the free energies of the complex, receptor, and ligand through the frames used in the calculation. In our approach, we used the whole trajectory (a total of 1000 frames). Different energy terms can be calculated according to Equations 2 to 6 as follows:

$\Delta Gbinding = \Delta H - T\Delta S$ (2)

$\Delta H = \Delta Egas + \Delta Esol$ (3)

$\Delta Egas = \Delta Eele + \Delta EvdW$ (4)

$\Delta Esolv = EGB + ESA$ (5)

$ESA = \gamma.SASA$ (6)

where ΔH is the enthalpy which can be calculated from gas-phase energy (E_{gas}) and solvation-free energy (E_{sol}). TΔS is the entropy contribution to the free binding energy. E_{gas} is composed of electrostatic and van der Waals terms; E_{ele}, E_{vdW}, respectively. E_{sol} can be calculated from the polar solvation energy (E_{GB}) and nonpolar solvation energy (E_{SA}) which is estimated from the solvent-accessible surface area.

Density Function Theory (DFT) calculations

The Gaussian 09 program was used to perform the quantum chemistry calculations using the DFT method. GaussianView5 was used to display all of the data files. The density function theory (DFT) at 6-311G++(d,p) basis set/B3LYP approach was utilized to optimize organic chemical structure of the compound under investigation and Chem3D 15.0 software was used to create the original chemical structures. Both the Total Electron Density (TED) and the Electrostatic Surface (ESP) maps were examined at the same theoretical level. GaussSum3.0 software was used to compute and evaluate the total density of state (TDOS) for the optimized log file.

Equations of Koopmans' theory: The chemical potential (μ), maximal charge acceptance (ΔN_{max}), global hardness (η), energy change (ΔE), electronegativity (χ), the global softness (σ), electrophilicity index (ω), ionization potential (IP), and electron affinity (EA)

$IP = -E_{HOMO}$

$EA = -E_{LUMO}$

$\mu= (IP + EA)/2$

$\eta= (IP - EA)$

$\chi= - \eta$

$\omega= \mu^2/(2 \eta)$

$\sigma= 1/ \eta$

$\Delta N= -(\mu/\eta)$

$\Delta E = -\omega$

$E_{gap} = E_{LUMO} – E_{HOMO}$

In silico ADMET studies

ADMET descriptors (absorption, distribution, metabolism, excretion and toxicity) of the compounds were determined using Discovery studio 4.0. At first, the CHARMM force field was applied then the compounds were prepared and minimized according to the preparation of small molecule protocol. Then, ADMET descriptors protocol was applied to carry out these studies.

In silico toxicity studies

The toxicity parameters of the synthesized compounds were calculated using Discovery studio 4.0. sorafenib was used as a reference drug. At first, the CHARMM force field was applied then the compounds were prepared and minimized according to the preparation of small molecule protocol. Then, different parameters were calculated from the toxicity prediction (extensible) protocol.

Biological testing

1- Mammalian cell lines culture

MCF-7 and HCT 116 cell lines were cultured on DMEM media. The cultured media were supplemented with 200 mM L-glutamine, 10.0% fetal bovine serum (Lonza), and 1.0% penicillin/streptomycin. Cells were seeded into 25.0 cm tissue culture flasks and incubated at 37 °C in a 5.0% CO_2 incubator for 24 h or till confluency.

2- **Safety assay**

The safety profiles of the tested compounds were checked on one non-cancerous cell line (W138) to determine the treatments concentrations that do not depict toxic effects against the tested cells. A portion of 100.0 μL of 6×10^4 cell/mL cells was seeded into each well of a 96-well plate and then the plates were incubated at 37 °C in a humidified 5.0% CO_2 incubator for 24 h. At the end of incubation period, the exhausted medium was replaced with 100.0 μL of different concentrations of the designated treatment (prepared in RPMI medium starting from 1.0 mM). The inoculated plates were incubated at the same growth conditions for another 24 h. At the end of incubation, cellular viability was assessed using MTS assay kit (Promega) according to the manual instruction.

***3- I*n vitro anticancer activity**

Anticancer activities of the tested compounds against MCF-7 and HCT 116 cell lines were quantified using MTS assay kit (Promega) as described by the Manufacturer.

4- Selectivity index (SI)

The selectivity index values of the tested compounds on cancer cells were calculated as described by Koch et al., with slight modifications; $SI=IC_{50}nc/IC_{50}cc$, where $IC_{50}nc$: the IC_{50} value of the tested compound on normal cells and $IC_{50}cc$: IC_{50} of the tested compound on cancer cell line.

5- In vitro VEGFR-2 kinase inhibitory assay

The IC_{50} of the tested derivative was determined against VEGFR-2 kinase with enzyme linked immunosorbent assay (ELISA). We used a human VEGFR-2 ELISA kit. VEGFR-2 specific antibody was seeded on a 96 well microplate and 100 μL of solution of the standard or the tested compound was added. After incubation for 2.5 h at room temperature and washing, 100 μL of prepared biotin antibody was added. Incubation of the plates for 1 h at room temperature and then washing were carried out before addition of streptavidin solution (100 μL). The mixture was incubated for 1 h at room temperature and then washed. After that, 100

μL of tetramethybenzidine (TMB) substrate solution was added before incubation for half an hour at room temperature. Finally, 50 μL of the stop solution was added immediately before the reading at 450 nm. The standard curve was then drawn; the concentration was on the X axis and the absorbance was on the Y axis.

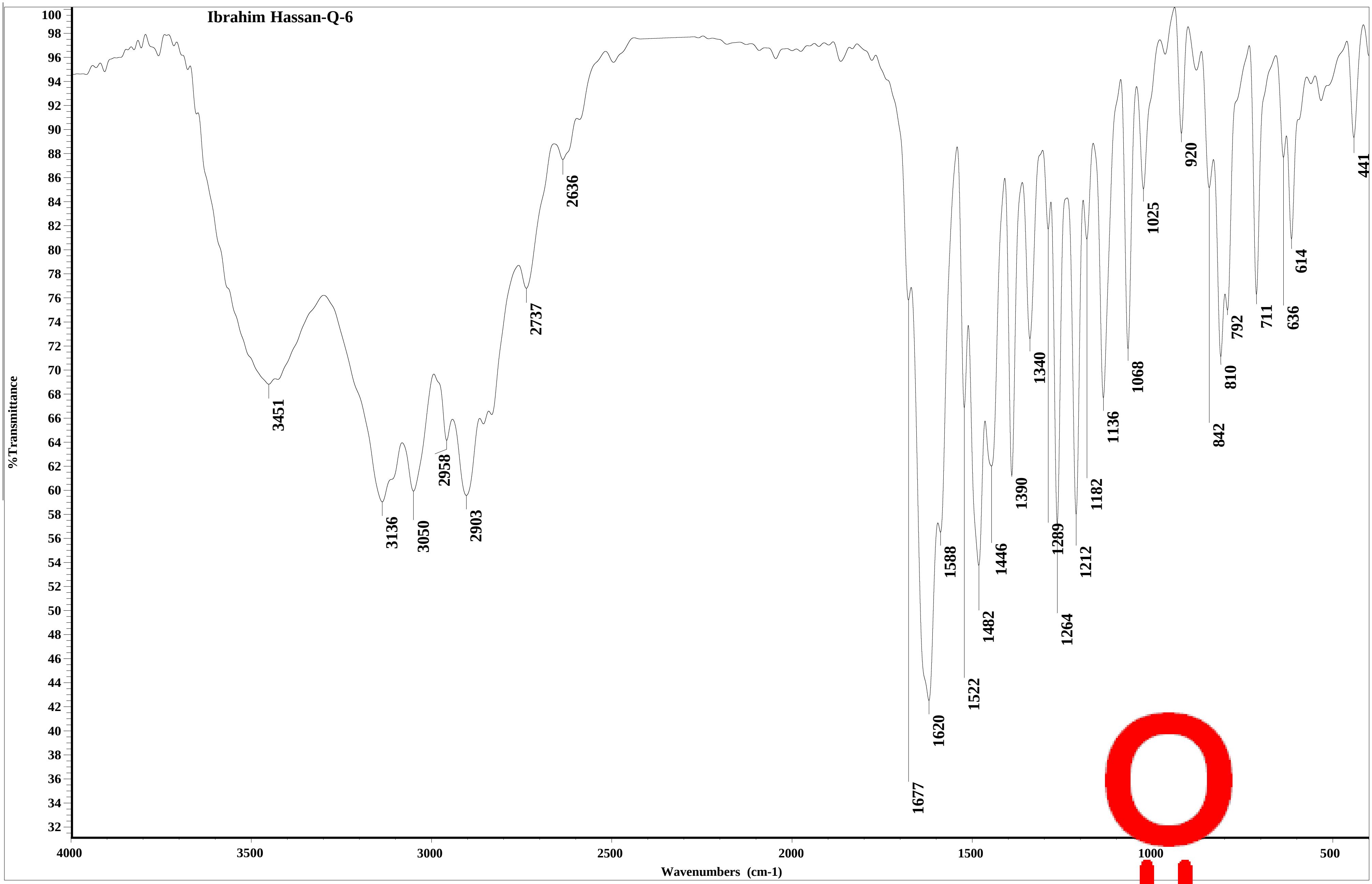

Number of sample scans: 32
Number of background scans: 32
Resolution: 8.000
Sample gain: 1.0
Optical velocity: 0.4747
Aperture: 150.00

Thermo Fisher SCIENTIFIC

Mon Jun 20 11:59:34 2022 (GMT+02:00)

Mansoura University
Faculty of Science
Spectral Analysis Unit
unitofspectra@gmail.com

1H NMR compound 10

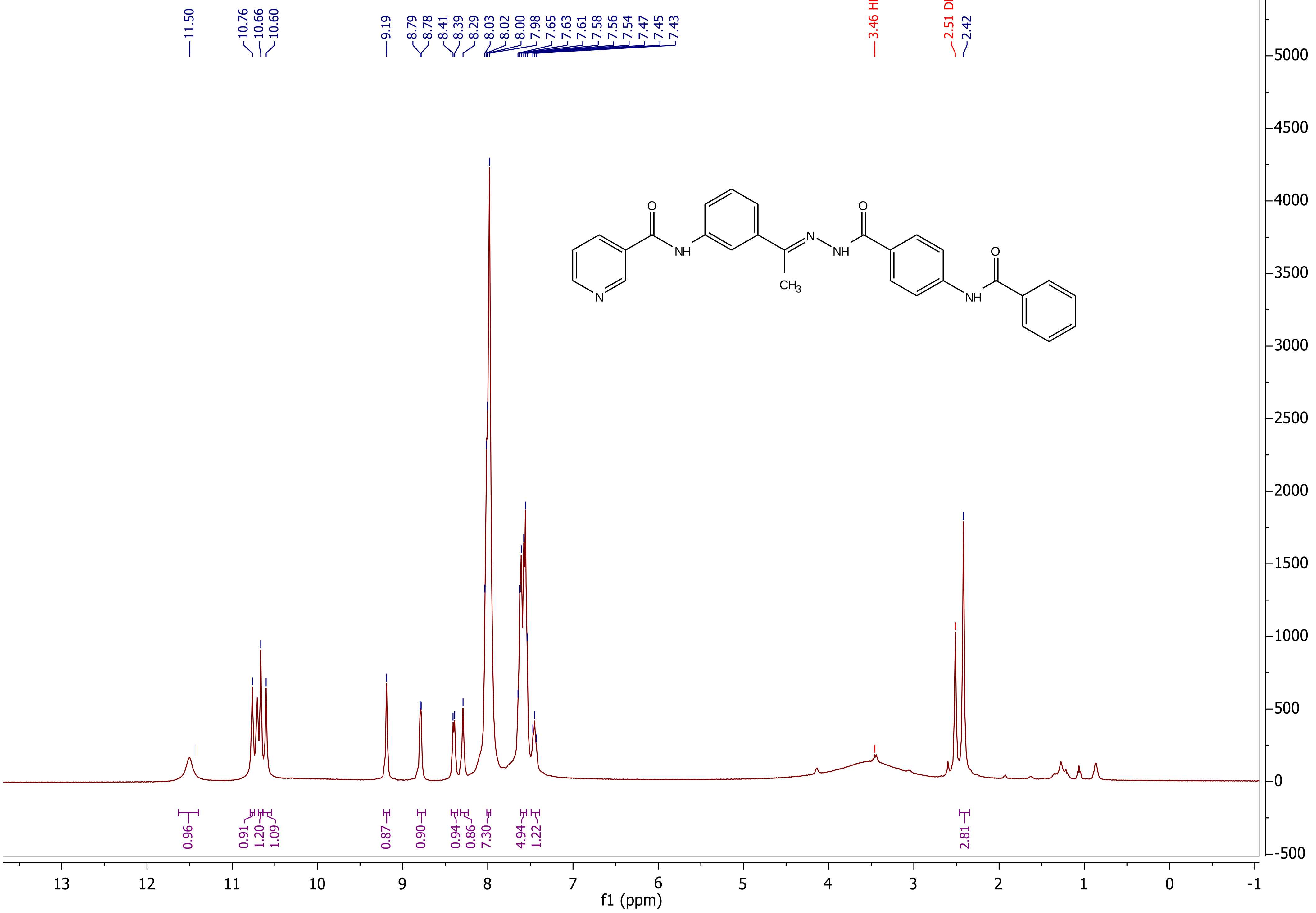

1H NMR compound 10

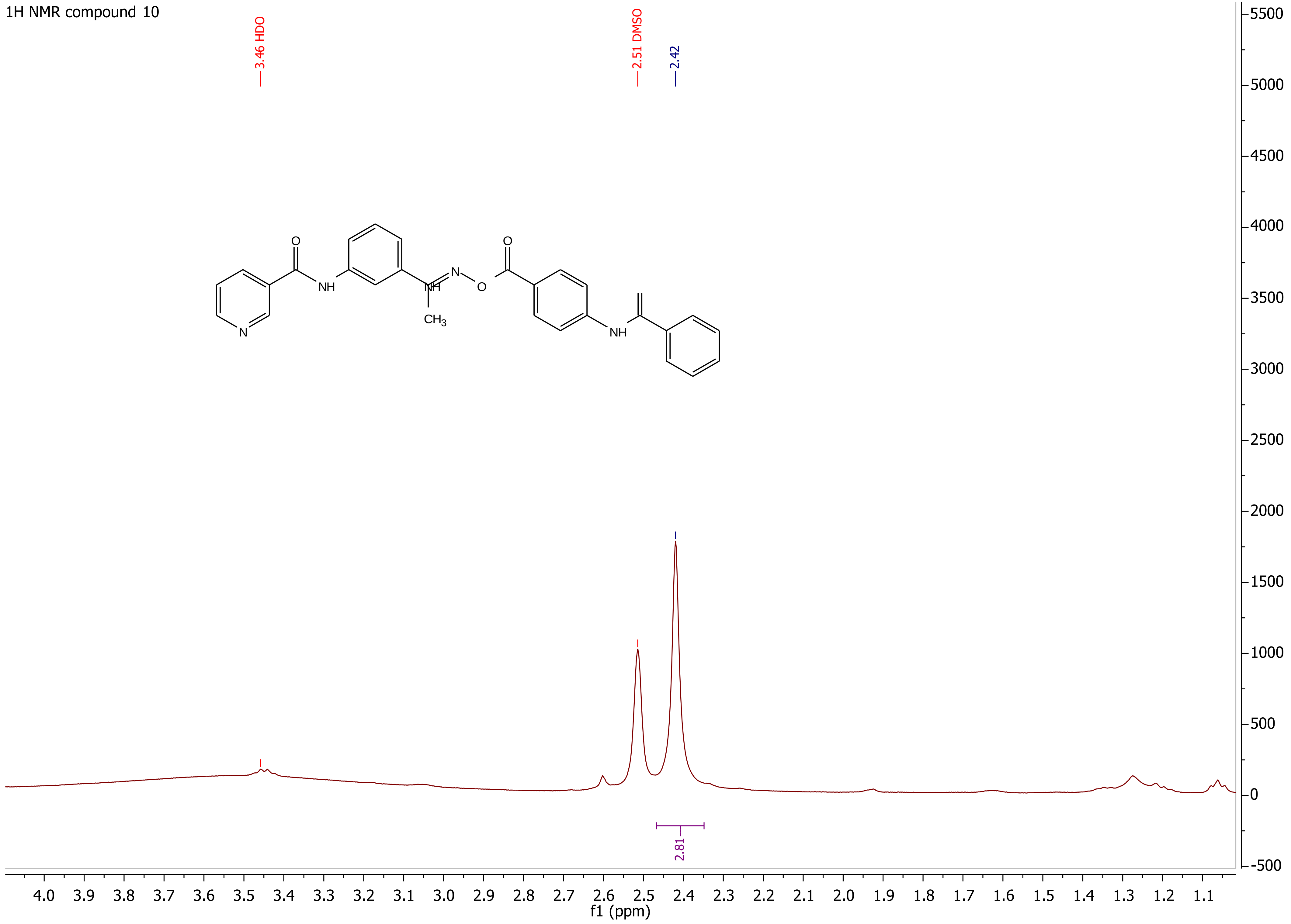

1H NMR compound 10

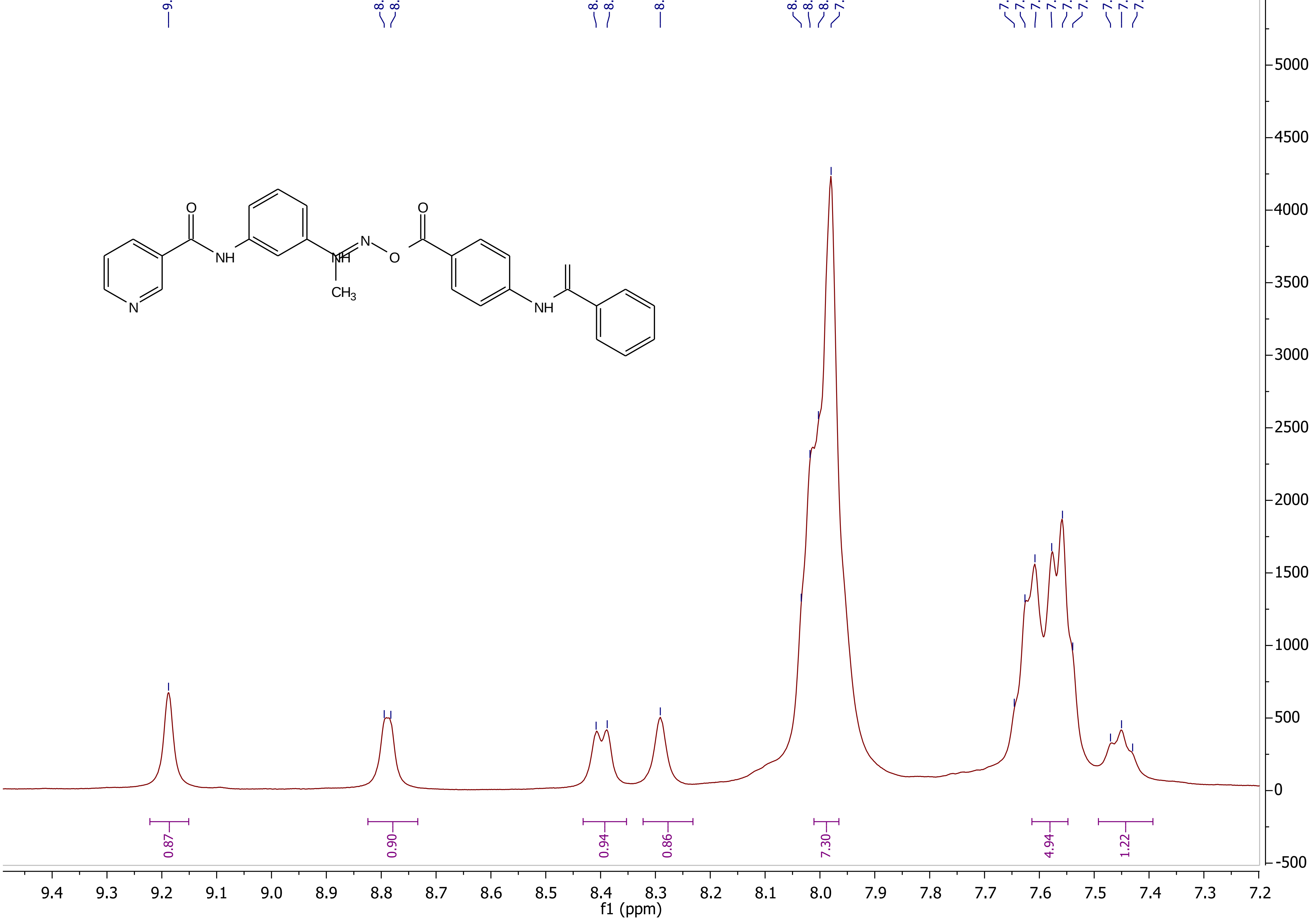

13C NMR compound 10

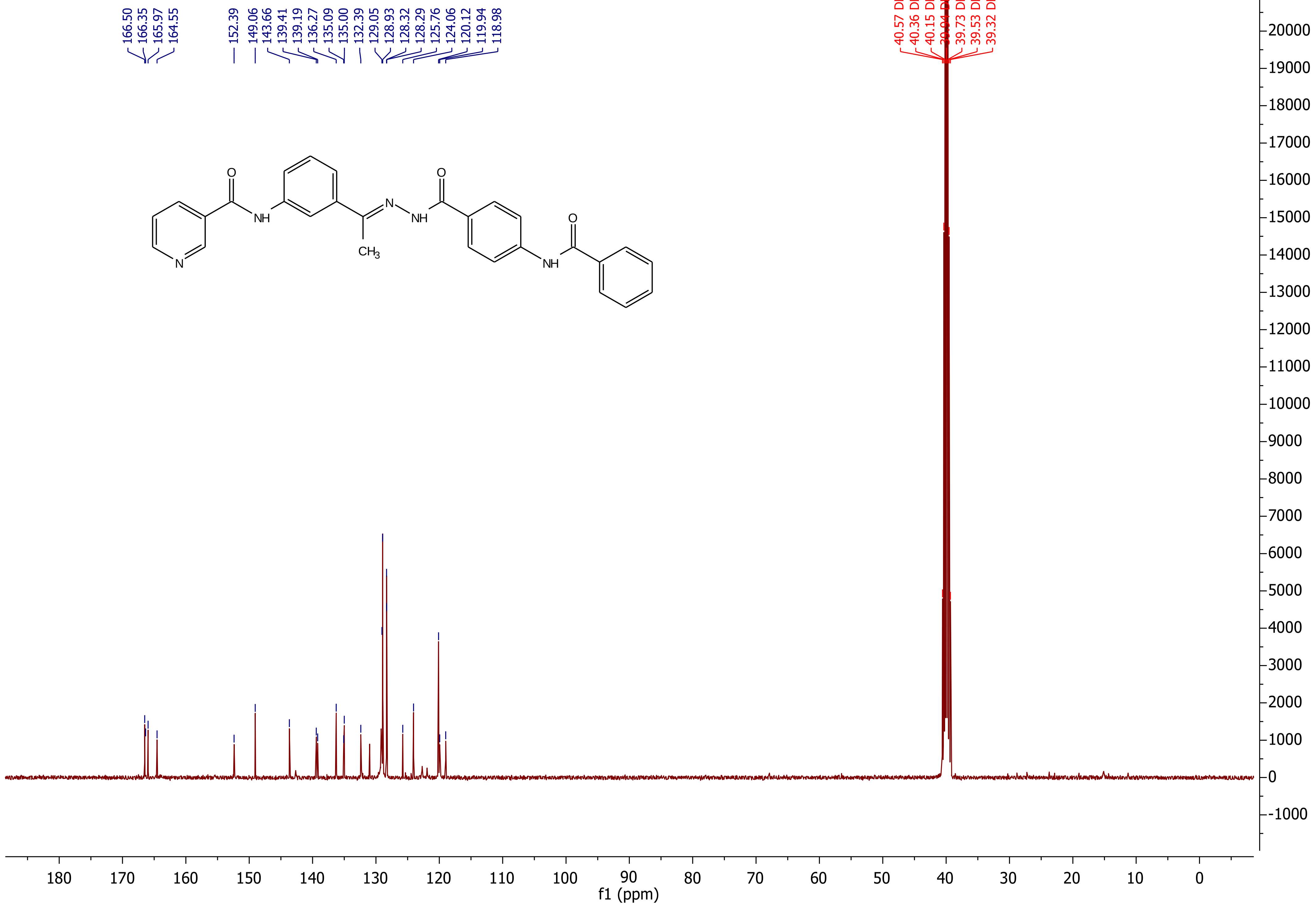

13C NMR compound 10

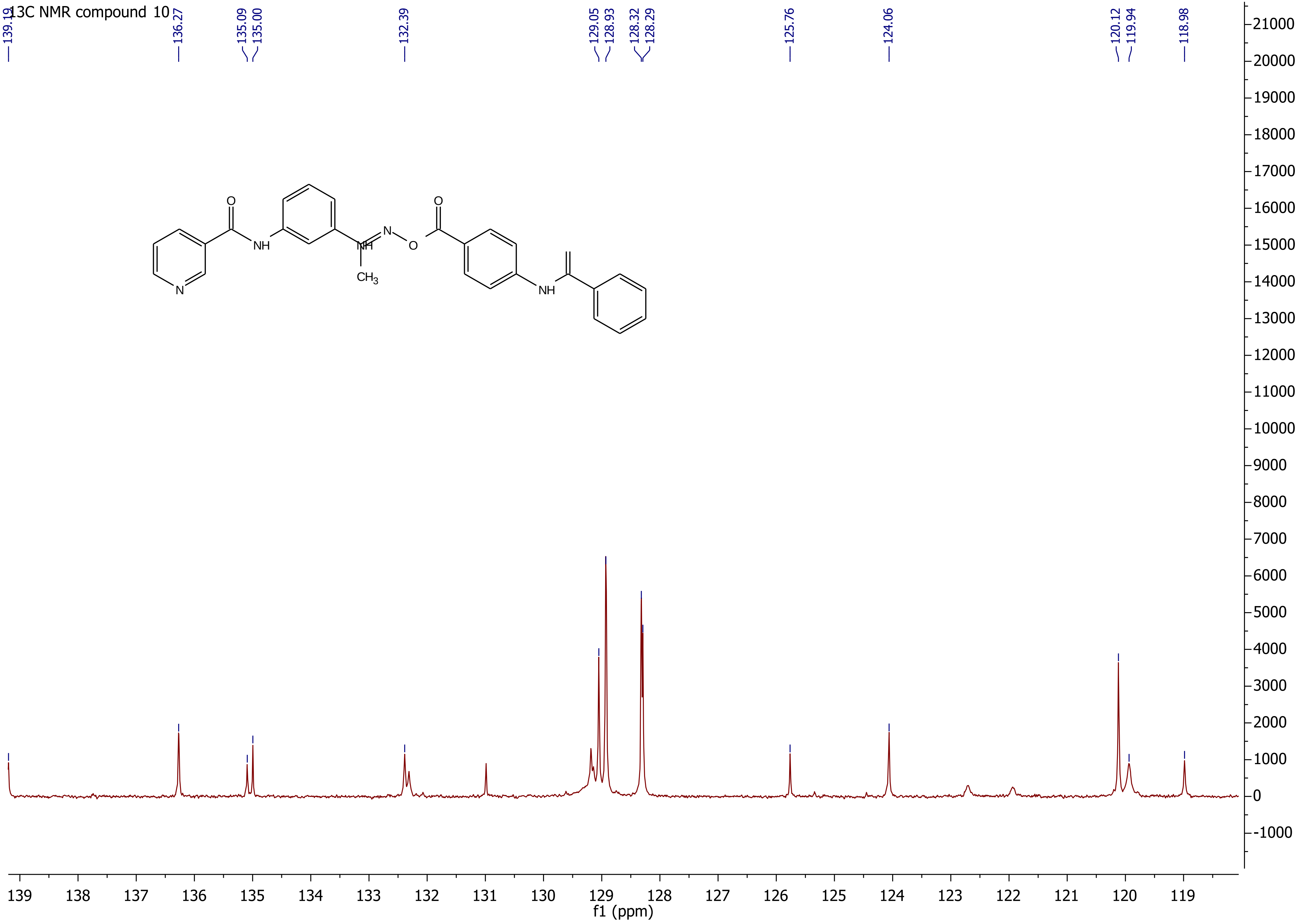

13C NMR compound 10

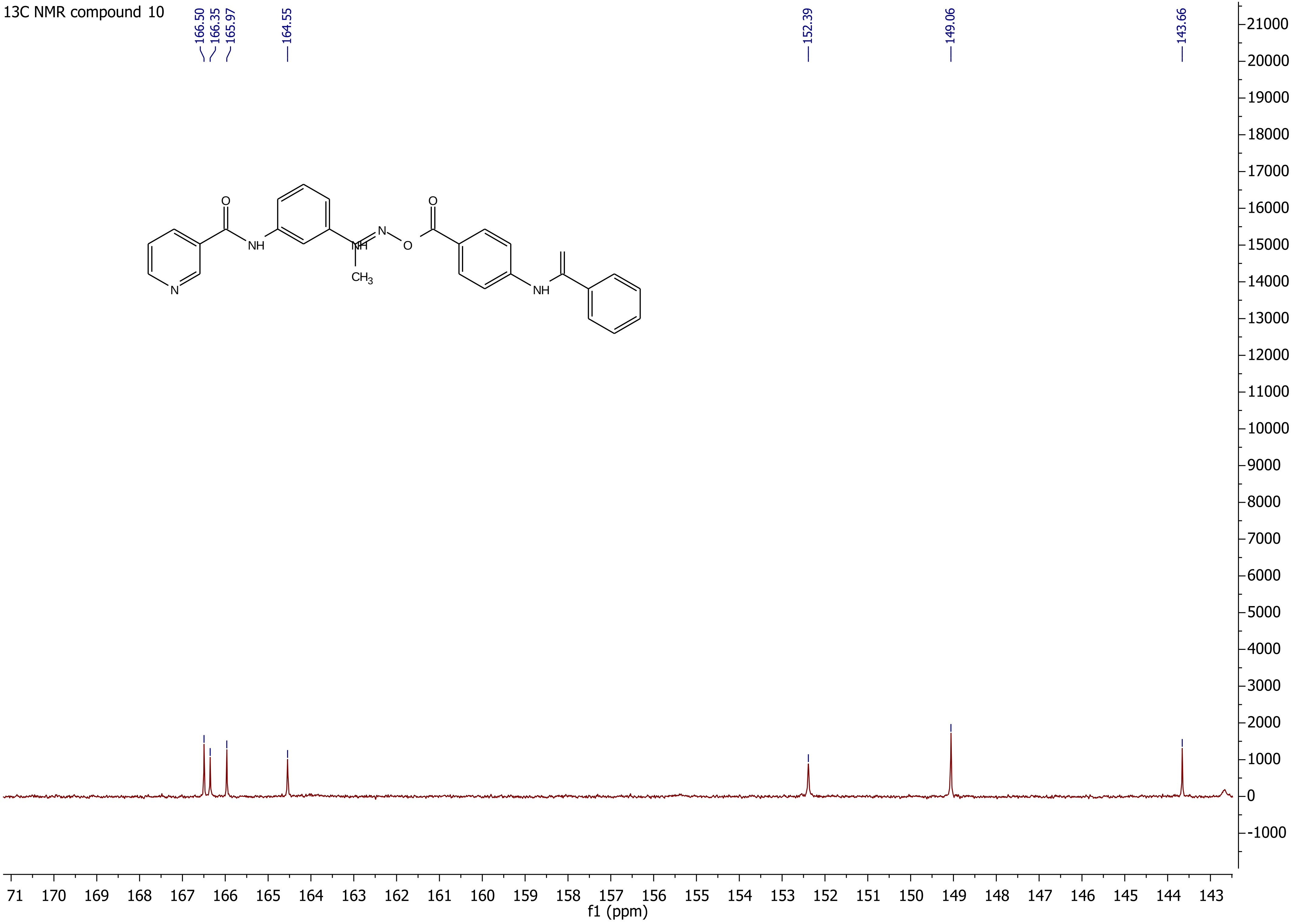

Compound 10

TOPKAT_Ames_Mutagenicity

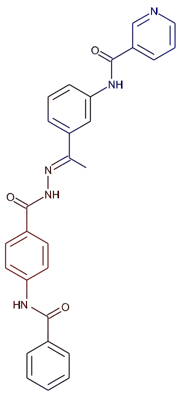

$C_{28}H_{23}N_5O_3$
Molecular Weight: 477.51391
ALogP: 3.082
Rotatable Bonds: 7
Acceptors: 5
Donors: 3

Model Prediction

Prediction: Non-Mutagen

Probability: 0.687

Enrichment: 1.23

Bayesian Score: -2.27

Mahalanobis Distance: 8.29

Mahalanobis Distance p-value: 0.976

Prediction: Positive if the Bayesian score is above the estimated best cutoff value from minimizing the false positive and false negative rate.
Probability: The esimated probability that the sample is in the positive category. This assumes that the Bayesian score follows a normal distribution and is different from the prediction using a cutoff.
Enrichment: An estimate of enrichment, that is, the increased likelihood (versus random) of this sample being in the category.
Bayesian Score: The standard Laplacian-modified Bayesian score.
Mahalanobis Distance: The Mahalanobis distance (MD) is the distance to the center of the training data. The larger the MD, the less trustworthy the prediction.
Mahalanobis Distance p-value: The p-value gives the fraction of training data with an MD greater than or equal to the one for the given sample, assuming normally distributed data. The smaller the p-value, the less trustworthy the prediciton. For highly non-normal X properties (e.g., fingerprints), the MD p-value is wildly inaccurate.

Structural Similar Compounds

Name	GLYBURIDE	6724-53-4	GLIPIZIDE
Structure	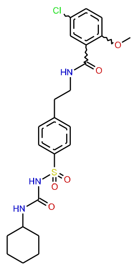	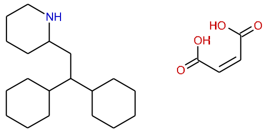	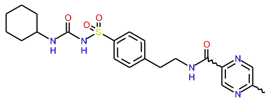
Actual Endpoint	Non-Mutagen	Non-Mutagen	Non-Mutagen
Predicted Endpoint	Non-Mutagen	Non-Mutagen	Non-Mutagen
Distance	0.593	0.616	0.633
Reference	PDR 1994	Kazius et. al., J. Med. Chem. (2005) 48, 312-320	PDR 1994

Model Applicability

Unknown features are fingerprint features in the query molecule, but not found or appearing too infrequently in the training set.

1. All properties and OPS components are within expected ranges.

Feature Contribution

Top features for positive contribution

Fingerprint	Bit/Smiles	Feature Structure	Score	Mutagen in training set
SCFP_12	555539852	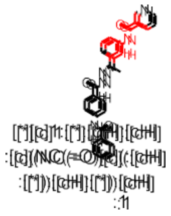	0.447	22 out of 24

SCFP_12	818445224	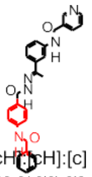 [*][c]1:[cH]:[cH]:[c](NC(=O)[c](:[*]):[*]):[cH]:[cH]:1	0.434	12 out of 13
SCFP_12	2096901122	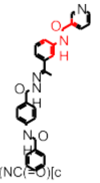 [*]:[cH]:[c](NC(=O)[c](:[*]):[*]):[cH]:[*]	0.429	33 out of 37

Top Features for negative contribution

Fingerprint	Bit/Smiles	Feature Structure	Score	Mutagen in training set
SCFP_12	391452900	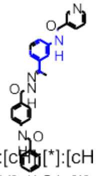 [*]N[c]1:[cH]:[*]:[cH]:[c](:[cH]:1)C(=[*])[*]	-0.916	1 out of 7
SCFP_12	1165971455	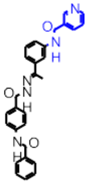 [*]NC(=O)[c]1:[cH]:[cH]:[cH]:n:[cH]:1	-0.762	0 out of 2
SCFP_12	903335088	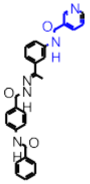 [*]NC(=O)[c]1:[cH]:[cH]:[*]:n:[cH]:1	-0.762	0 out of 2

Sorafenib

TOPKAT_Ames_Mutagenicity

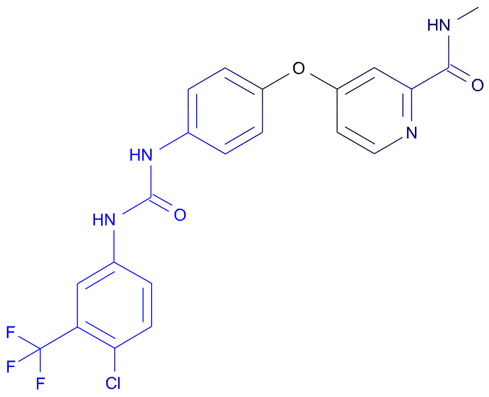

$C_{21}H_{16}ClF_3N_4O_3$
Molecular Weight: 464.82494
ALogP: 4.175
Rotatable Bonds: 6
Acceptors: 4
Donors: 3

Structural Similar Compounds

Name	GLYBURIDE	38914-96-4	93957-54-1
Structure	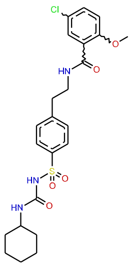	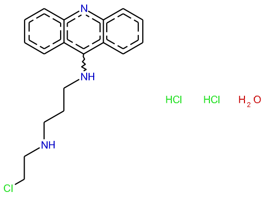	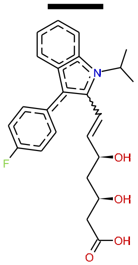
Actual Endpoint	Non-Mutagen	Mutagen	Non-Mutagen
Predicted Endpoint	Non-Mutagen	Mutagen	Non-Mutagen
Distance	0.590	0.592	0.600
Reference	PDR 1994	Kazius et. al., J. Med. Chem. (2005) 48, 312-320	US Environmental Protection Agency at http://www.epa.gov/NCCT/dsstox/sdf_isscan_external.html

Model Prediction

Prediction: Non-Mutagen

Probability: 0.0531

Enrichment: 0.0951

Bayesian Score: -19.7

Mahalanobis Distance: 13.1

Mahalanobis Distance p-value: 2.73e-006

Prediction: Positive if the Bayesian score is above the estimated best cutoff value from minimizing the false positive and false negative rate.
Probability: The esimated probability that the sample is in the positive category. This assumes that the Bayesian score follows a normal distribution and is different from the prediction using a cutoff.
Enrichment: An estimate of enrichment, that is, the increased likelihood (versus random) of this sample being in the category.
Bayesian Score: The standard Laplacian-modified Bayesian score.
Mahalanobis Distance: The Mahalanobis distance (MD) is the distance to the center of the training data. The larger the MD, the less trustworthy the prediction.
Mahalanobis Distance p-value: The p-value gives the fraction of training data with an MD greater than or equal to the one for the given sample, assuming normally distributed data. The smaller the p-value, the less trustworthy the prediciton. For highly non-normal X properties (e.g., fingerprints), the MD p-value is wildly inaccurate.

Model Applicability

Unknown features are fingerprint features in the query molecule, but not found or appearing too infrequently in the training set.

1. All properties and OPS components are within expected ranges.

Feature Contribution

Top features for positive contribution				
**Fingerprint**	**Bit/Smiles**	**Feature Structure**	**Score**	**Mutagen in training set**
SCFP_12	-347281112	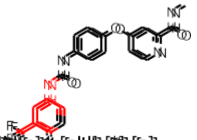 [*]N[c]1:[cH]:[*]:[c](([*]):[c](:[cH]:1)C([*])([*])[*]	0.337	18 out of 22

SCFP_12	1208843554	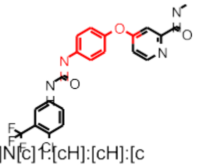 [*]N[c]1:[cH]:[cH]:[c](O[c](:[*]):[*]):[cH]:[cH]:1	0.337	6 out of 7
SCFP_12	-1943080297	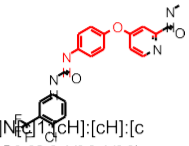 [*]N[c]1:[cH]:[cH]:[c](O[c]2:[cH]:[cH]:[*]:[c]([*]):[cH]:2):[cH]:[cH]:1	0.304	5 out of 6

Top Features for negative contribution

Fingerprint	Bit/Smiles	Feature Structure	Score	Mutagen in training set
SCFP_12	816802409	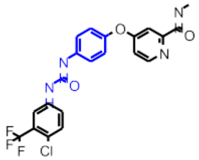 [*]NC(=O)N[c]1:[cH]:[cH]:[c]([*]):[cH]:[cH]:1	-1.82	0 out of 9
SCFP_12	-300280774	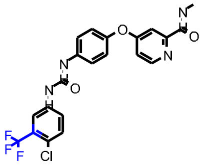 [*]:[c](:[*])C(F)(F)F	-1.51	3 out of 30
SCFP_12	-1903175541	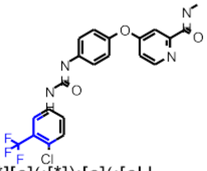 [*][c](:[*]):[c](:[cH]:[*])C(F)(F)F	-1.51	3 out of 30

Compound 10

TOPKAT_Developmental_Toxicity_Potential

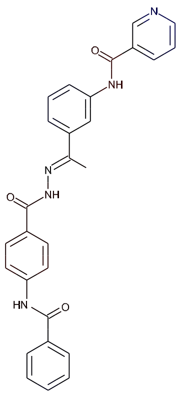

$C_{28}H_{23}N_5O_3$

Molecular Weight: 477.51391

ALogP: 3.082

Rotatable Bonds: 7

Acceptors: 5

Donors: 3

Structural Similar Compounds

Name	Ochratoxin a	Amsacrine	Citreoviridin
Structure	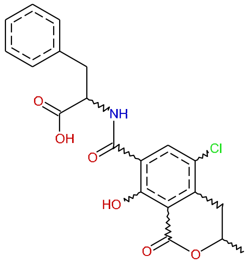	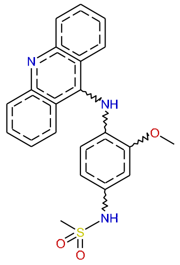	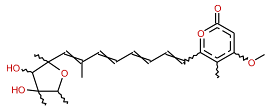
Actual Endpoint	Toxic	Toxic	Toxic
Predicted Endpoint	Toxic	Toxic	Toxic
Distance	0.636	0.669	0.681
Reference	Toxicol Appl Pharmacol 37(2):331-8; 1976	Fundam Appl Toxicol 7(2):214-20; 1986	Food Chem Toxicol 24(12):1315-20; 1986

Model Applicability

Unknown features are fingerprint features in the query molecule, but not found or appearing too infrequently in the training set.

1. OPS PC17 out of range. Value: 2.9151. Training min, max, SD, explained variance: -2.7025, 2.8536, 1.067, 0.0167.

Model Prediction

Prediction: Non-Toxic

Probability: 0.506

Enrichment: 0.963

Bayesian Score: -1.12

Mahalanobis Distance: 9.15

Mahalanobis Distance p-value: 0.136

Prediction: Positive if the Bayesian score is above the estimated best cutoff value from minimizing the false positive and false negative rate.
Probability: The esimated probability that the sample is in the positive category. This assumes that the Bayesian score follows a normal distribution and is different from the prediction using a cutoff.
Enrichment: An estimate of enrichment, that is, the increased likelihood (versus random) of this sample being in the category.
Bayesian Score: The standard Laplacian-modified Bayesian score.
Mahalanobis Distance: The Mahalanobis distance (MD) is the distance to the center of the training data. The larger the MD, the less trustworthy the prediction.
Mahalanobis Distance p-value: The p-value gives the fraction of training data with an MD greater than or equal to the one for the given sample, assuming normally distributed data. The smaller the p-value, the less trustworthy the prediciton. For highly non-normal X properties (e.g., fingerprints), the MD p-value is wildly inaccurate.

Feature Contribution

Top features for positive contribution

Fingerprint	Bit/Smiles	Feature Structure	Score	Toxic in training set
SCFP_6	282594097	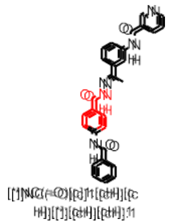	0.441	3 out of 3

SCFP_6	1257084377	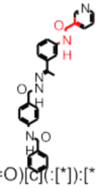 [*]NC(=O)[c](:[*]):[*]	0.362	14 out of 18
SCFP_6	903335088	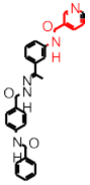 [*]NC(=O)[c]1:[cH]:[c H]:[*]:n:[cH]:1	0.271	1 out of 1

Top Features for negative contribution

Fingerprint	Bit/Smiles	Feature Structure	Score	Toxic in training set
SCFP_6	-758850909	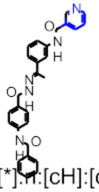 [*][c]1:[*]:n:[cH]:[c H]:[cH]:1	-0.646	2 out of 9
SCFP_6	-937094999	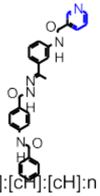 [*]1:[cH]:[cH]:[cH]:n :[cH]:1	-0.358	3 out of 9
SCFP_6	-496201075	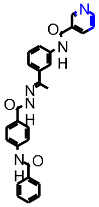 [*]:[cH]:[cH]:n:[*]	-0.289	8 out of 21

Sorafenib

TOPKAT_Developmental_Toxicity_Potential

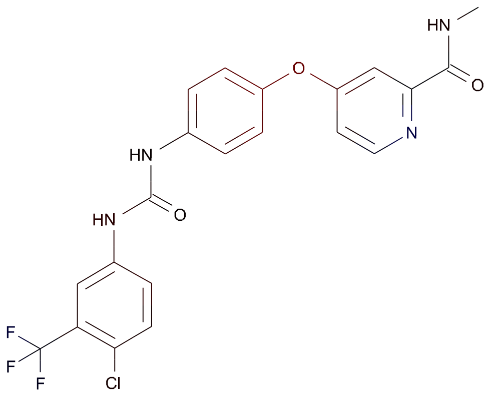

$C_{21}H_{16}ClF_3N_4O_3$
Molecular Weight: 464.82494
ALogP: 4.175
Rotatable Bonds: 6
Acceptors: 4
Donors: 3

Model Prediction

Prediction: Toxic

Probability: 0.592

Enrichment: 1.13

Bayesian Score: 1.15

Mahalanobis Distance: 12.6

Mahalanobis Distance p-value: 2.07e-006

Prediction: Positive if the Bayesian score is above the estimated best cutoff value from minimizing the false positive and false negative rate.
Probability: The esimated probability that the sample is in the positive category. This assumes that the Bayesian score follows a normal distribution and is different from the prediction using a cutoff.
Enrichment: An estimate of enrichment, that is, the increased likelihood (versus random) of this sample being in the category.
Bayesian Score: The standard Laplacian-modified Bayesian score.
Mahalanobis Distance: The Mahalanobis distance (MD) is the distance to the center of the training data. The larger the MD, the less trustworthy the prediction.
Mahalanobis Distance p-value: The p-value gives the fraction of training data with an MD greater than or equal to the one for the given sample, assuming normally distributed data. The smaller the p-value, the less trustworthy the prediciton. For highly non-normal X properties (e.g., fingerprints), the MD p-value is wildly inaccurate.

Structural Similar Compounds

Name	Chenodiol	Amsacrine	Ochratoxin a
Structure	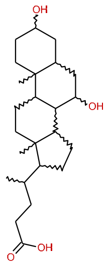	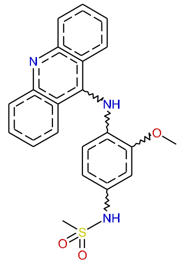	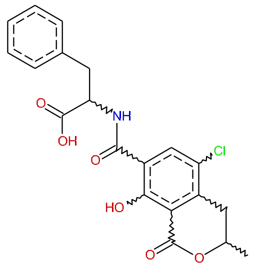
Actual Endpoint	Toxic	Toxic	Toxic
Predicted Endpoint	Toxic	Toxic	Toxic
Distance	0.631	0.637	0.644
Reference	Arch Int Pharm 246:149-158; 1980	Fundam Appl Toxicol 7(2):214-20; 1986	Toxicol Appl Pharmacol 37(2):331-8; 1976

Model Applicability

Unknown features are fingerprint features in the query molecule, but not found or appearing too infrequently in the training set.

1. All properties and OPS components are within expected ranges.

Feature Contribution

Top features for positive contribution				
**Fingerprint**	**Bit/Smiles**	**Feature Structure**	**Score**	**Toxic in training set**
SCFP_6	1559190850	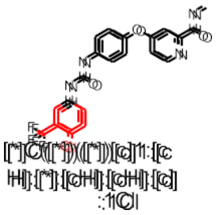	0.441	3 out of 3

SCFP_6	-488587948	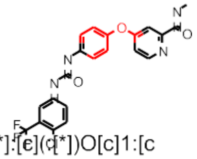 [*]:[c](c[*])O[c]1:[cH]:[cH]:[*]:[cH]:[cH]:1	0.381	2 out of 2
SCFP_6	-347281112	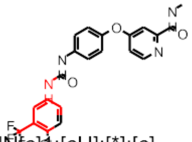 [*]N[c]1:[cH]:[*]:[c]([*]):[c](:[cH]:1)C([*])([*])[*]	0.381	2 out of 2
**Top Features for negative contribution**				
**Fingerprint**	**Bit/Smiles**	**Feature Structure**	**Score**	**Toxic in training set**
SCFP_6	-1794974220	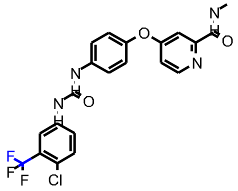 [*]C([*])([*])F	-0.55	2 out of 8
SCFP_6	-937094999	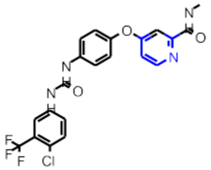 [*]1:[cH]:[cH]:[cH]:n:[cH]:1	-0.358	3 out of 9
SCFP_6	-496201075	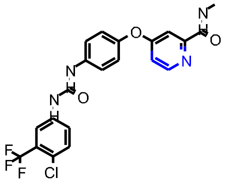 [*]:[cH]:[cH]:n:[*]	-0.289	8 out of 21

Compound 10

TOPKAT_Mouse_Female_FDA_None_vs_Carcinogen

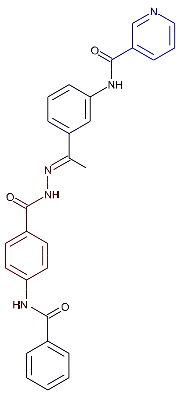

$C_{28}H_{23}N_5O_3$

Molecular Weight: 477.51391

ALogP: 3.082

Rotatable Bonds: 7

Acceptors: 5

Donors: 3

Structural Similar Compounds

Name	Glimepride	Glyburide	Bicalutamide
Structure	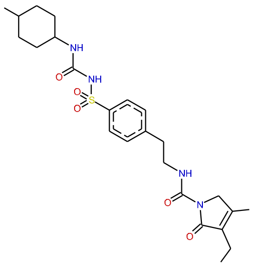	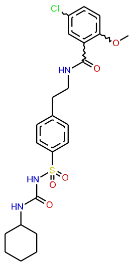	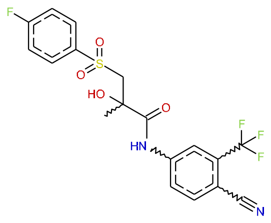
Actual Endpoint	Carcinogen	Non-Carcinogen	Non-Carcinogen
Predicted Endpoint	Carcinogen	Non-Carcinogen	Non-Carcinogen
Distance	0.551	0.586	0.633
Reference	US FDA (Centre for Drug Eval.& Res./Off. Testing & Res.) Sept. 1997	US FDA (Centre for Drug Eval.& Res./Off. Testing & Res.) Sept. 1997	US FDA (Centre for Drug Eval.& Res./Off. Testing & Res.) Sept. 1997

Model Prediction

Prediction: Non-Carcinogen

Probability: 0.236

Enrichment: 0.737

Bayesian Score: -1.58

Mahalanobis Distance: 13.3

Mahalanobis Distance p-value: 0.000166

Prediction: Positive if the Bayesian score is above the estimated best cutoff value from minimizing the false positive and false negative rate.
Probability: The esimated probability that the sample is in the positive category. This assumes that the Bayesian score follows a normal distribution and is different from the prediction using a cutoff.
Enrichment: An estimate of enrichment, that is, the increased likelihood (versus random) of this sample being in the category.
Bayesian Score: The standard Laplacian-modified Bayesian score.
Mahalanobis Distance: The Mahalanobis distance (MD) is the distance to the center of the training data. The larger the MD, the less trustworthy the prediction.
Mahalanobis Distance p-value: The p-value gives the fraction of training data with an MD greater than or equal to the one for the given sample, assuming normally distributed data. The smaller the p-value, the less trustworthy the prediciton. For highly non-normal X properties (e.g., fingerprints), the MD p-value is wildly inaccurate.

Model Applicability

Unknown features are fingerprint features in the query molecule, but not found or appearing too infrequently in the training set.

1. All properties and OPS components are within expected ranges.
2. Unknown ECFP_2 feature: 128986386: [*]\N=C(/C)\[c](:[*]):[*]
3. Unknown ECFP_2 feature: 560380707: [*]NN=C([*])[*]

Feature Contribution

Top features for positive contribution

Fingerprint	Bit/Smiles	Feature Structure	Score	Carcinogen in training set
ECFP_6	-1087070950	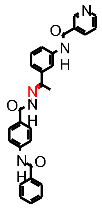 [*]N=[*]	0.724	10 out of 14

ECFP_6	738938915	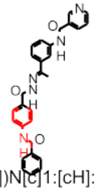 [*]C(=[*])N[c]1:[cH]:[cH]:[*]:[cH]:[cH]:1	0.617	2 out of 2
ECFP_6	544048674	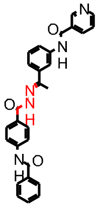 [*]C(=[*])NN=[*]	0.617	2 out of 2
**Top Features for negative contribution**				
**Fingerprint**	**Bit/Smiles**	**Feature Structure**	**Score**	**Carcinogen in training set**
ECFP_6	2013347047	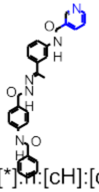 [*][c]1:[*]:n:[cH]:[cH]:[cH]:1	-0.805	0 out of 4
ECFP_6	2007300961	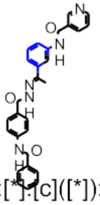 [*][c]1:[*]:[c]([*]):[cH]:[cH]:[cH]:1	-0.652	5 out of 34
ECFP_6	-1818873508	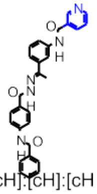 [*][c]1:[cH]:[cH]:[cH]:n:[cH]:1	-0.482	0 out of 2

Sorafenib

TOPKAT_Mouse_Female_FDA_None_vs_Carcinogen

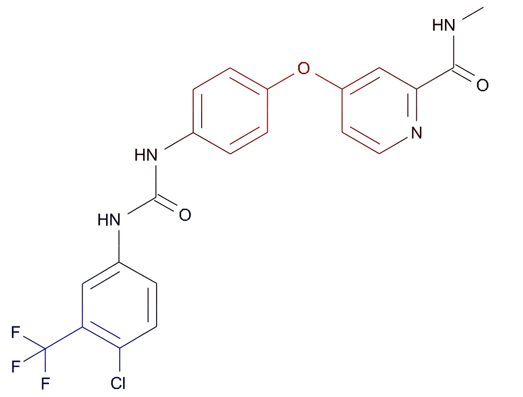

$C_{21}H_{16}ClF_3N_4O_3$
Molecular Weight: 464.82494
ALogP: 4.175
Rotatable Bonds: 6
Acceptors: 4
Donors: 3

Structural Similar Compounds

Name	Glimepride	Glyburide	Fluvastatin
Structure	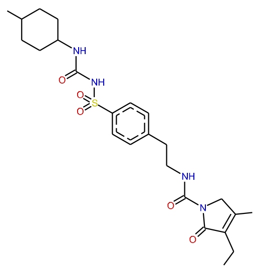	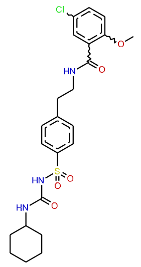	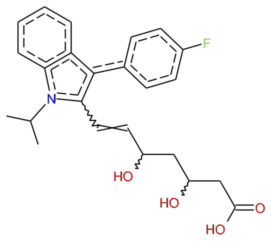
Actual Endpoint	Carcinogen	Non-Carcinogen	Non-Carcinogen
Predicted Endpoint	Carcinogen	Non-Carcinogen	Non-Carcinogen
Distance	0.605	0.615	0.625
Reference	US FDA (Centre for Drug Eval.& Res./Off. Testing & Res.) Sept. 1997	US FDA (Centre for Drug Eval.& Res./Off. Testing & Res.) Sept. 1997	US FDA (Centre for Drug Eval.& Res./Off. Testing & Res.) Sept. 1997

Model Prediction

Prediction: Carcinogen

Probability: 0.257

Enrichment: 0.801

Bayesian Score: -0.321

Mahalanobis Distance: 14.9

Mahalanobis Distance p-value: 4.21e-007

Prediction: Positive if the Bayesian score is above the estimated best cutoff value from minimizing the false positive and false negative rate.
Probability: The esimated probability that the sample is in the positive category. This assumes that the Bayesian score follows a normal distribution and is different from the prediction using a cutoff.
Enrichment: An estimate of enrichment, that is, the increased likelihood (versus random) of this sample being in the category.
Bayesian Score: The standard Laplacian-modified Bayesian score.
Mahalanobis Distance: The Mahalanobis distance (MD) is the distance to the center of the training data. The larger the MD, the less trustworthy the prediction.
Mahalanobis Distance p-value: The p-value gives the fraction of training data with an MD greater than or equal to the one for the given sample, assuming normally distributed data. The smaller the p-value, the less trustworthy the prediciton. For highly non-normal X properties (e.g., fingerprints), the MD p-value is wildly inaccurate.

Model Applicability

Unknown features are fingerprint features in the query molecule, but not found or appearing too infrequently in the training set.

1. OPS PC20 out of range. Value: -3.3309. Training min, max, SD, explained variance: -3.1862, 4.4571, 1.28, 0.0167.

Feature Contribution

Top features for positive contribution

Fingerprint	Bit/Smiles	Feature Structure	Score	Carcinogen in training set
ECFP_6	738938915	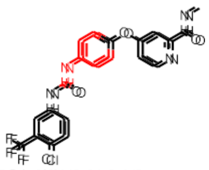 [*]C(=[*])N[c]1:[cH]:[cH]:[*]:[cH]:[cH]:1	0.617	2 out of 2

ECFP_6	1338334141	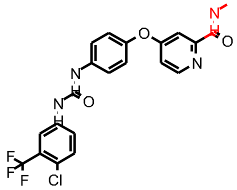 [*]C(=[*])NC	0.442	2 out of 3
ECFP_6	1305253718	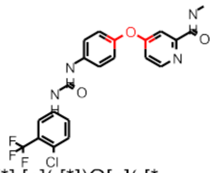 [*]:[c](:[*])O[c](:[*]):[*]	0.424	1 out of 1
**Top Features for negative contribution**				
**Fingerprint**	**Bit/Smiles**	**Feature Structure**	**Score**	**Carcinogen in training set**
ECFP_6	1335691903	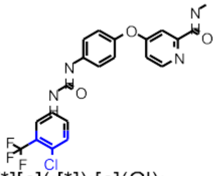 [*][c](:[*]):[c](Cl):[cH]:[*]	-0.669	3 out of 22
ECFP_6	1336678434	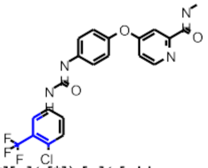 [*][c](:[*]):[c](:[cH]:[*])C([*])([*])[*]	-0.657	0 out of 3
ECFP_6	-1952889961	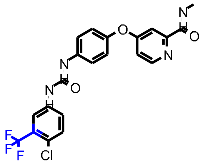 [*]:[c](:[*])C(F)(F)F	-0.657	0 out of 3

Sorafenib

TOPKAT_Mouse_Female_FDA_Single_vs_Multiple

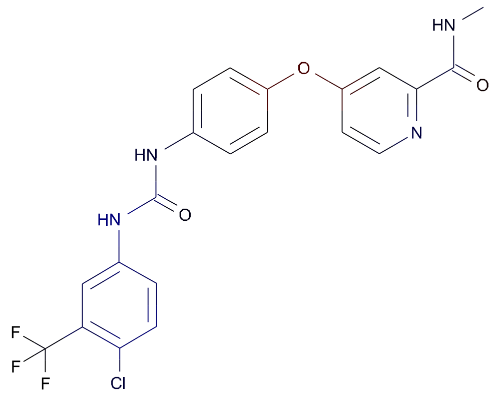

$C_{21}H_{16}ClF_3N_4O_3$
Molecular Weight: 464.82494

ALogP: 4.175

Rotatable Bonds: 6

Acceptors: 4

Donors: 3

Structural Similar Compounds

Name	Glimepride	Labetalol	Lansoprazole
Structure	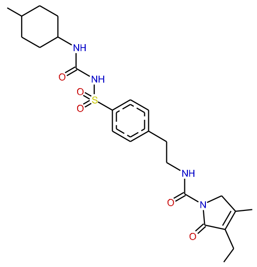	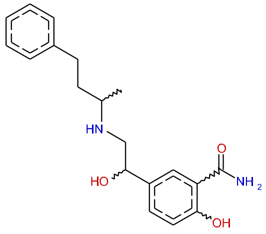	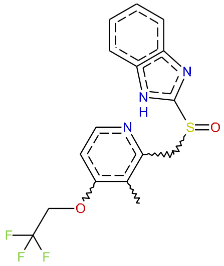
Actual Endpoint	Single-Carcinogen	Single-Carcinogen	Single-Carcinogen
Predicted Endpoint	Single-Carcinogen	Single-Carcinogen	Single-Carcinogen
Distance	0.599	0.808	0.820
Reference	US FDA (Centre for Drug Eval.& Res./Off. Testing & Res.) Sept. 1997	US FDA (Centre for Drug Eval.& Res./Off. Testing & Res.) Sept. 1997	US FDA (Centre for Drug Eval.& Res./Off. Testing & Res.) Sept. 1997

Model Prediction

Prediction: Single-Carcinogen

Probability: 0.283

Enrichment: 0.691

Bayesian Score: -3.89

Mahalanobis Distance: 11.1

Mahalanobis Distance p-value: 0.00221

Prediction: Positive if the Bayesian score is above the estimated best cutoff value from minimizing the false positive and false negative rate.
Probability: The esimated probability that the sample is in the positive category. This assumes that the Bayesian score follows a normal distribution and is different from the prediction using a cutoff.
Enrichment: An estimate of enrichment, that is, the increased likelihood (versus random) of this sample being in the category.
Bayesian Score: The standard Laplacian-modified Bayesian score.
Mahalanobis Distance: The Mahalanobis distance (MD) is the distance to the center of the training data. The larger the MD, the less trustworthy the prediction.
Mahalanobis Distance p-value: The p-value gives the fraction of training data with an MD greater than or equal to the one for the given sample, assuming normally distributed data. The smaller the p-value, the less trustworthy the prediciton. For highly non-normal X properties (e.g., fingerprints), the MD p-value is wildly inaccurate.

Model Applicability

Unknown features are fingerprint features in the query molecule, but not found or appearing too infrequently in the training set.

1. All properties and OPS components are within expected ranges.
2. Unknown ECFP_2 feature: 1336678434: [*][c](:[*]):[c](C([*])([*])[*]):c:[*]
3. Unknown ECFP_2 feature: -1952889961: [*]:[c](:[*])C(F)(F)F

Feature Contribution

Top features for positive contribution

Fingerprint	Bit/Smiles	Feature Structure	Score	Multiple-Carcinogen in training set
ECFP_4	-834094296	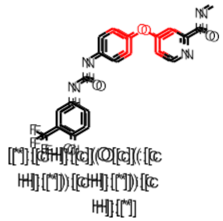	0.351	1 out of 1

ECFP_4	1407472008	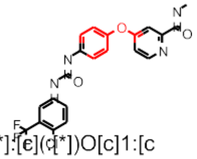 [*]:[c](:[*])O[c]1:[cH]:[cH]:[*]:[cH]:[cH]:1	0.351	1 out of 1
ECFP_4	143734695	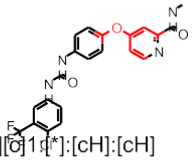 [*][c]1:[*]:[cH]:[cH]:[c](O[c](:[*]):[*]):[cH]:1	0.351	1 out of 1

Top Features for negative contribution

Fingerprint	Bit/Smiles	Feature Structure	Score	Multiple-Carcinogen in training set
ECFP_4	888054369	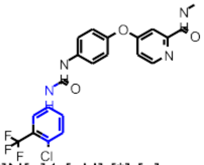 [*]N[c]1:[cH]:[*]:[c]([*]):[cH]:[cH]:1	-0.8	0 out of 3
ECFP_4	1335691903	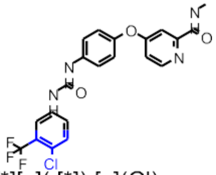 [*][c](:[*]):[c](Cl):[cH]:[*]	-0.8	0 out of 3
ECFP_4	1338334141	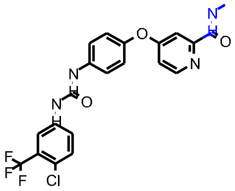 [*]C(=[*])NC	-0.597	0 out of 2

Compound 10

TOPKAT_Mouse_Male_FDA_None_vs_Carcinogen

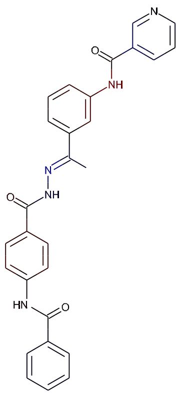

$C_{28}H_{23}N_5O_3$

Molecular Weight: 477.51391

ALogP: 3.082

Rotatable Bonds: 7

Acceptors: 5

Donors: 3

Model Prediction

Prediction: Carcinogen

Probability: 0.27

Enrichment: 0.917

Bayesian Score: -1.41

Mahalanobis Distance: 13.9

Mahalanobis Distance p-value: 1.01e-005

Prediction: Positive if the Bayesian score is above the estimated best cutoff value from minimizing the false positive and false negative rate.
Probability: The esimated probability that the sample is in the positive category. This assumes that the Bayesian score follows a normal distribution and is different from the prediction using a cutoff.
Enrichment: An estimate of enrichment, that is, the increased likelihood (versus random) of this sample being in the category.
Bayesian Score: The standard Laplacian-modified Bayesian score.
Mahalanobis Distance: The Mahalanobis distance (MD) is the distance to the center of the training data. The larger the MD, the less trustworthy the prediction.
Mahalanobis Distance p-value: The p-value gives the fraction of training data with an MD greater than or equal to the one for the given sample, assuming normally distributed data. The smaller the p-value, the less trustworthy the prediciton. For highly non-normal X properties (e.g., fingerprints), the MD p-value is wildly inaccurate.

Structural Similar Compounds

Name	Glimepride	Glyburide	Bicalutamide
Structure	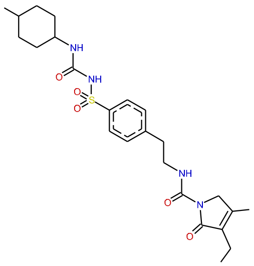	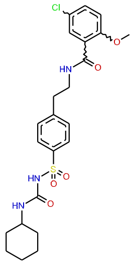	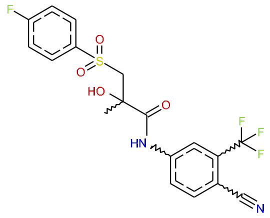
Actual Endpoint	Carcinogen	Non-Carcinogen	Carcinogen
Predicted Endpoint	Carcinogen	Non-Carcinogen	Carcinogen
Distance	0.539	0.583	0.610
Reference	US FDA (Centre for Drug Eval.& Res./Off. Testing & Res.) Sept. 1997	US FDA (Centre for Drug Eval.& Res./Off. Testing & Res.) Sept. 1997	US FDA (Centre for Drug Eval.& Res./Off. Testing & Res.) Sept. 1997

Model Applicability

Unknown features are fingerprint features in the query molecule, but not found or appearing too infrequently in the training set.

1. All properties and OPS components are within expected ranges.

Feature Contribution

Top features for positive contribution

Fingerprint	Bit/Smiles	Feature Structure	Score	Carcinogen in training set
FCFP_6	-581879738	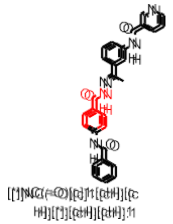	0.77	4 out of 5

FCFP_6	-1838187238	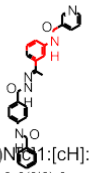 [*]C(=[*])N[c]1:[cH]:[cH]:[*]:[c]([*]):[cH]:1	0.565	4 out of 7
FCFP_6	1294255210	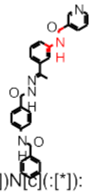 [*]C(=[*])N[c](:[*]):[*]	0.441	12 out of 28
**Top Features for negative contribution**				
**Fingerprint**	**Bit/Smiles**	**Feature Structure**	**Score**	**Carcinogen in training set**
FCFP_6	-885520711	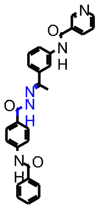 [*]C(=[*])NN=[*]	-0.839	0 out of 5
FCFP_6	1153798395	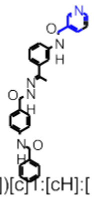 [*]C(=[*])[c]1:[cH]:[*]:[cH]:n:[cH]:1	-0.582	0 out of 3
FCFP_6	-1549192822	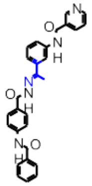 [*]\N=C(/C)\[c](:[*]):[*]	-0.489	3 out of 21

Sorafenib

TOPKAT_Mouse_Male_FDA_None_vs_Carcinogen

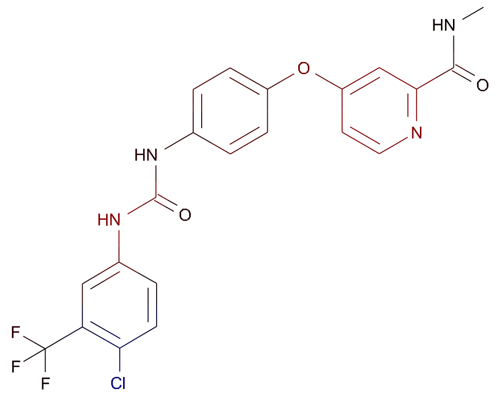

$C_{21}H_{16}ClF_3N_4O_3$

Molecular Weight: 464.82494

ALogP: 4.175

Rotatable Bonds: 6

Acceptors: 4

Donors: 3

Structural Similar Compounds

Name	Glyburide	Glimepride	Fluvastatin
Structure	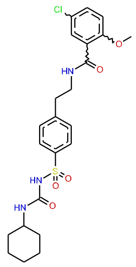	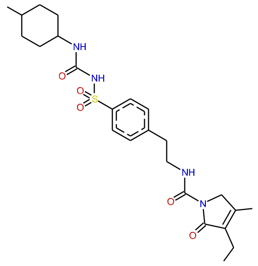	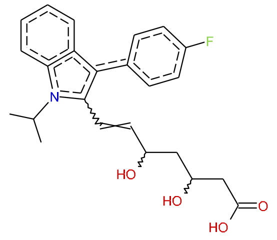
Actual Endpoint	Non-Carcinogen	Carcinogen	Non-Carcinogen
Predicted Endpoint	Non-Carcinogen	Carcinogen	Non-Carcinogen
Distance	0.594	0.599	0.603
Reference	US FDA (Centre for Drug Eval.& Res./Off. Testing & Res.) Sept. 1997	US FDA (Centre for Drug Eval.& Res./Off. Testing & Res.) Sept. 1997	US FDA (Centre for Drug Eval.& Res./Off. Testing & Res.) Sept. 1997

Model Prediction

Prediction: Carcinogen

Probability: 0.444

Enrichment: 1.51

Bayesian Score: 4.21

Mahalanobis Distance: 20.3

Mahalanobis Distance p-value: 1.28e-019

Prediction: Positive if the Bayesian score is above the estimated best cutoff value from minimizing the false positive and false negative rate.
Probability: The esimated probability that the sample is in the positive category. This assumes that the Bayesian score follows a normal distribution and is different from the prediction using a cutoff.
Enrichment: An estimate of enrichment, that is, the increased likelihood (versus random) of this sample being in the category.
Bayesian Score: The standard Laplacian-modified Bayesian score.
Mahalanobis Distance: The Mahalanobis distance (MD) is the distance to the center of the training data. The larger the MD, the less trustworthy the prediction.
Mahalanobis Distance p-value: The p-value gives the fraction of training data with an MD greater than or equal to the one for the given sample, assuming normally distributed data. The smaller the p-value, the less trustworthy the prediciton. For highly non-normal X properties (e.g., fingerprints), the MD p-value is wildly inaccurate.

Model Applicability

Unknown features are fingerprint features in the query molecule, but not found or appearing too infrequently in the training set.

1. All properties and OPS components are within expected ranges.

Feature Contribution

Top features for positive contribution

Fingerprint	Bit/Smiles	Feature Structure	Score	Carcinogen in training set
FCFP_6	71953198	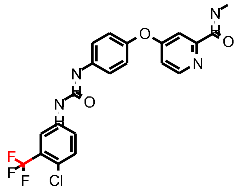 [*]C([*])([*])F	0.612	12 out of 23

FCFP_6	-1838187238	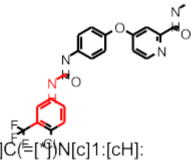 [*]C(=[*])N[c]1:[cH]:[cH]:[*]:[c]([*]):[cH]:1	0.565	4 out of 7
FCFP_6	140656626	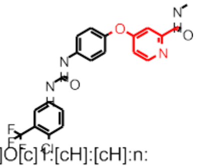 [*]O[c]1:[cH]:[cH]:n:[c](:[cH]:1)C(=[*])[*]	0.46	1 out of 1

Top Features for negative contribution

Fingerprint	Bit/Smiles	Feature Structure	Score	Carcinogen in training set
FCFP_6	2104062943	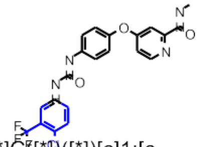 [*]C([*])([*])[c]1:[cH]:[*]:[cH]:[cH]:[c]:1Cl	-1.01	1 out of 17
FCFP_6	551850122	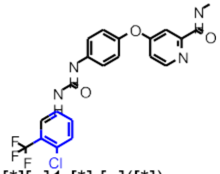 [*][c]1:[*]:[c]([*]):[c](Cl):[cH]:[cH]:1	-0.433	8 out of 49
FCFP_6	71476542	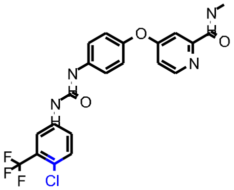 [*]:[c](:[*])Cl	-0.406	10 out of 59

Compound 10

TOPKAT_Mouse_Male_FDA_Single_vs_Multiple

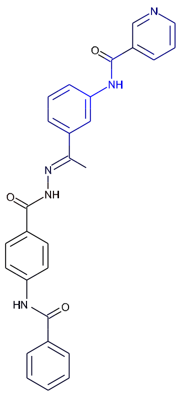

$C_{28}H_{23}N_5O_3$
Molecular Weight: 477.51391
ALogP: 3.082
Rotatable Bonds: 7
Acceptors: 5
Donors: 3

Model Prediction

Prediction: Single-Carcinogen
Probability: 0.154
Enrichment: 0.513
Bayesian Score: -12.4
Mahalanobis Distance: 15.6
Mahalanobis Distance p-value: 9.06e-007

Prediction: Positive if the Bayesian score is above the estimated best cutoff value from minimizing the false positive and false negative rate.
Probability: The esimated probability that the sample is in the positive category. This assumes that the Bayesian score follows a normal distribution and is different from the prediction using a cutoff.
Enrichment: An estimate of enrichment, that is, the increased likelihood (versus random) of this sample being in the category.
Bayesian Score: The standard Laplacian-modified Bayesian score.
Mahalanobis Distance: The Mahalanobis distance (MD) is the distance to the center of the training data. The larger the MD, the less trustworthy the prediction.
Mahalanobis Distance p-value: The p-value gives the fraction of training data with an MD greater than or equal to the one for the given sample, assuming normally distributed data. The smaller the p-value, the less trustworthy the prediciton. For highly non-normal X properties (e.g., fingerprints), the MD p-value is wildly inaccurate.

Structural Similar Compounds

Name	Glimepride	Bicalutamide	Primidolol
Structure	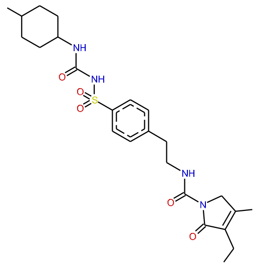	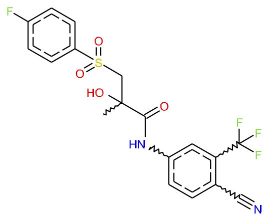	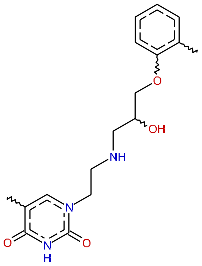
Actual Endpoint	Single-Carcinogen	Single-Carcinogen	Single-Carcinogen
Predicted Endpoint	Single-Carcinogen	Single-Carcinogen	Single-Carcinogen
Distance	0.551	0.680	0.796
Reference	US FDA (Centre for Drug Eval.& Res./Off. Testing & Res.) Sept. 1997	US FDA (Centre for Drug Eval.& Res./Off. Testing & Res.) Sept. 1997	US FDA (Centre for Drug Eval.& Res./Off. Testing & Res.) Sept. 1997

Model Applicability

Unknown features are fingerprint features in the query molecule, but not found or appearing too infrequently in the training set.

1. All properties and OPS components are within expected ranges.
2. Unknown FCFP_2 feature: 581019816: [*]NN=C([*])[*]
3. Unknown FCFP_2 feature: -885520711: [*]C(=[*])NN=[*]

Feature Contribution

Top features for positive contribution

Fingerprint	Bit/Smiles	Feature Structure	Score	Multiple-Carcinogen in training set
FCFP_12	547884906	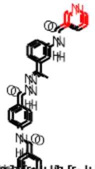 [*][c]1:[*][cH]:[cH]:n:[cH]:1	0.4	1 out of 1

FCFP_12	-581879738	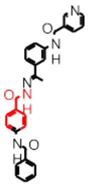 [*]NC(=O)[c]1:[cH]:[cH]:[*]:[cH]:[cH]:1	0.239	2 out of 4
FCFP_12	-1549103449	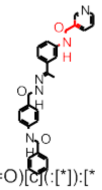 [*]NC(=O)[c](:[*]):[*]	0.168	3 out of 7
**Top Features for negative contribution**				
**Fingerprint**	**Bit/Smiles**	**Feature Structure**	**Score**	**Multiple-Carcinogen in training set**
FCFP_12	1294255210	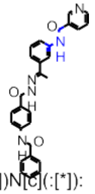 [*]C(=[*])N[c](:[*]):[*]	-1.63	0 out of 12
FCFP_12	590925877	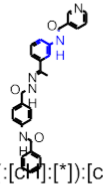 [*]N[c](:[cH]:[*]):[cH]:[*]	-0.998	1 out of 13
FCFP_12	-1838187238	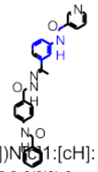 [*]C(=[*])N[c]1:[cH]:[cH]:[*]:[c]([*]):[cH]:1	-0.859	0 out of 4

Sorafenib

TOPKAT_Mouse_Male_FDA_Single_vs_Multiple

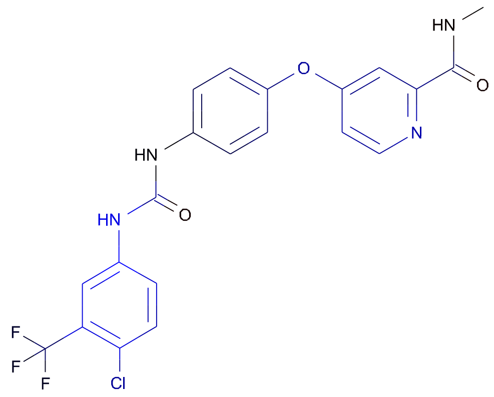

$C_{21}H_{16}ClF_3N_4O_3$
Molecular Weight: 464.82494
ALogP: 4.175
Rotatable Bonds: 6
Acceptors: 4
Donors: 3

Structural Similar Compounds

Name	Glimepride	Bicalutamide	Lansoprazole
Structure	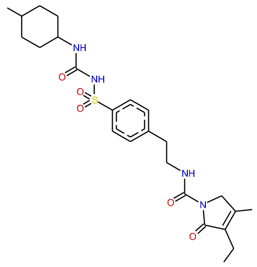	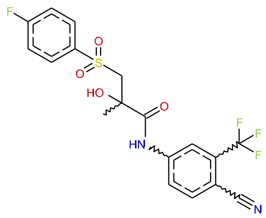	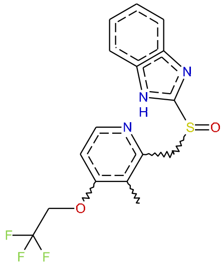
Actual Endpoint	Single-Carcinogen	Single-Carcinogen	Single-Carcinogen
Predicted Endpoint	Single-Carcinogen	Single-Carcinogen	Single-Carcinogen
Distance	0.626	0.700	0.866
Reference	US FDA (Centre for Drug Eval.& Res./Off. Testing & Res.) Sept. 1997	US FDA (Centre for Drug Eval.& Res./Off. Testing & Res.) Sept. 1997	US FDA (Centre for Drug Eval.& Res./Off. Testing & Res.) Sept. 1997

Model Prediction

Prediction: Single-Carcinogen

Probability: 0.139

Enrichment: 0.461

Bayesian Score: -14.7

Mahalanobis Distance: 21.3

Mahalanobis Distance p-value: 4.93e-011

Prediction: Positive if the Bayesian score is above the estimated best cutoff value from minimizing the false positive and false negative rate.
Probability: The esimated probability that the sample is in the positive category. This assumes that the Bayesian score follows a normal distribution and is different from the prediction using a cutoff.
Enrichment: An estimate of enrichment, that is, the increased likelihood (versus random) of this sample being in the category.
Bayesian Score: The standard Laplacian-modified Bayesian score.
Mahalanobis Distance: The Mahalanobis distance (MD) is the distance to the center of the training data. The larger the MD, the less trustworthy the prediction.
Mahalanobis Distance p-value: The p-value gives the fraction of training data with an MD greater than or equal to the one for the given sample, assuming normally distributed data. The smaller the p-value, the less trustworthy the prediciton. For highly non-normal X properties (e.g., fingerprints), the MD p-value is wildly inaccurate.

Model Applicability

Unknown features are fingerprint features in the query molecule, but not found or appearing too infrequently in the training set.

1. All properties and OPS components are within expected ranges.

Feature Contribution

Top features for positive contribution

Fingerprint	Bit/Smiles	Feature Structure	Score	Multiple-Carcinogen in training set
FCFP_12	1499521844	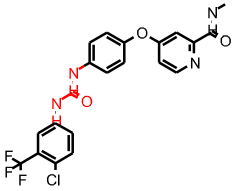 [*]NC(=O)N[*]	0.39	5 out of 9

FCFP_12	-904785030	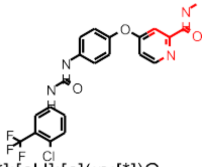 [*]:[cH]:[c](:n:[*])C (=O)NC	0.174	1 out of 2
FCFP_12	-1549103449	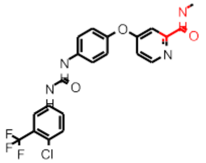 [*]NC(=O)[c](:[*]):[*]	0.168	3 out of 7

Top Features for negative contribution

Fingerprint	Bit/Smiles	Feature Structure	Score	Multiple-Carcinogen in training set
FCFP_12	1294255210	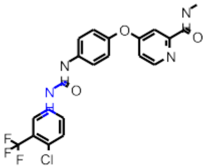 [*]C(=[*])N[c](:[*]): [*]	-1.63	0 out of 12
FCFP_12	590925877	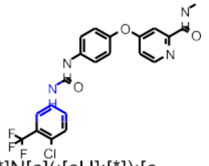 [*]N[c](:[cH]:[*]):[c H]:[*]	-0.998	1 out of 13
FCFP_12	-1462709112	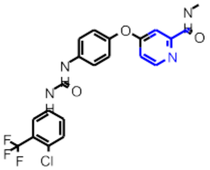 [*]C(=[*])[c]1:[cH]:[*]:[cH]:[cH]:n:1	-0.994	0 out of 5

Compound 10

TOPKAT_Ocular_Irritancy_Mild_vs_Moderate_Severe

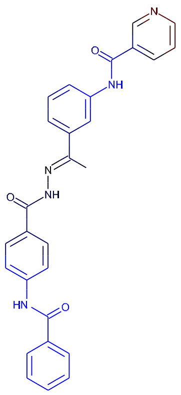

$C_{28}H_{23}N_5O_3$

Molecular Weight: 477.51391

ALogP: 3.082

Rotatable Bonds: 7

Acceptors: 5

Donors: 3

Model Prediction

Prediction: Mild

Probability: 0.0538

Enrichment: 0.0781

Bayesian Score: -11.9

Mahalanobis Distance: 7.9

Mahalanobis Distance p-value: 0.942

Prediction: Positive if the Bayesian score is above the estimated best cutoff value from minimizing the false positive and false negative rate.
Probability: The esimated probability that the sample is in the positive category. This assumes that the Bayesian score follows a normal distribution and is different from the prediction using a cutoff.
Enrichment: An estimate of enrichment, that is, the increased likelihood (versus random) of this sample being in the category.
Bayesian Score: The standard Laplacian-modified Bayesian score.
Mahalanobis Distance: The Mahalanobis distance (MD) is the distance to the center of the training data. The larger the MD, the less trustworthy the prediction.
Mahalanobis Distance p-value: The p-value gives the fraction of training data with an MD greater than or equal to the one for the given sample, assuming normally distributed data. The smaller the p-value, the less trustworthy the prediciton. For highly non-normal X properties (e.g., fingerprints), the MD p-value is wildly inaccurate.

Structural Similar Compounds

Name	4;4'-DIAMINO-1;1'-DIANTHRIMIDE	1-AMINO-4-BENZOYLAMINO-ANTHRAQUINONE	ANTHRAQUINONE; 1-((2-HYDROXYETHYL)AMINO)-4-(METHYLAMINO)-
Structure	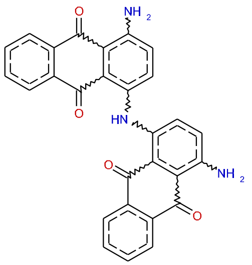	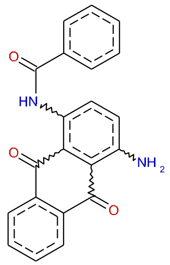	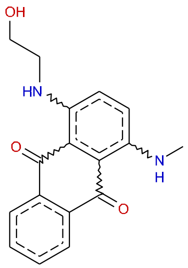
Actual Endpoint	Mild	Mild	Mild
Predicted Endpoint	Mild	Mild	Mild
Distance	0.726	0.786	0.798
Reference	28ZPAK-;125;72	28ZPAK-;124;72	28ZPAK 245;72

Model Applicability

Unknown features are fingerprint features in the query molecule, but not found or appearing too infrequently in the training set.

1. All properties and OPS components are within expected ranges.
2. Unknown FCFP_2 feature: 581019816: [*]NN=C([*])[*]

Feature Contribution

Top features for positive contribution

Fingerprint	Bit/Smiles	Feature Structure	Score	Moderate_Severe in training set
FCFP_10	547884906	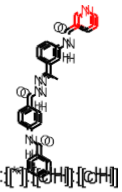 [*][c]1:[*]:[cH]:[cH]:n:[cH]:1	0.317	4 out of 4

FCFP_10	-1695756380	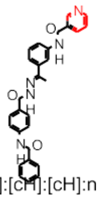 [*]1:[cH]:[cH]:[cH]:n:[cH]:1	0.285	10 out of 11
FCFP_10	-124655670	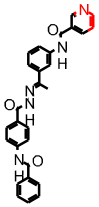 [*]:[cH]:[cH]:n:[*]	0.259	14 out of 16
**Top Features for negative contribution**				
**Fingerprint**	**Bit/Smiles**	**Feature Structure**	**Score**	**Moderate_Severe in training set**
FCFP_10	1175232969	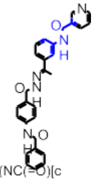 [*]:[cH]:[c](NC(=O)[c](:[*]):[*]):[cH]:[*]	-1.29	0 out of 4
FCFP_10	-1925475824	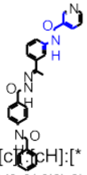 [*]:[cH]:[c]([illegible]cH]:[*])C(=O)N[c](:[*]):[*]	-1.29	0 out of 4
FCFP_10	-581879738	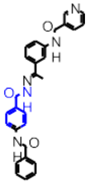 [*]NC(=O)[c]1:[cH]:[cH]:[*]:[cH]:[cH]:1	-1.29	0 out of 4

Sorafenib

TOPKAT_Ocular_Irritancy_Mild_vs_Moderate_Severe

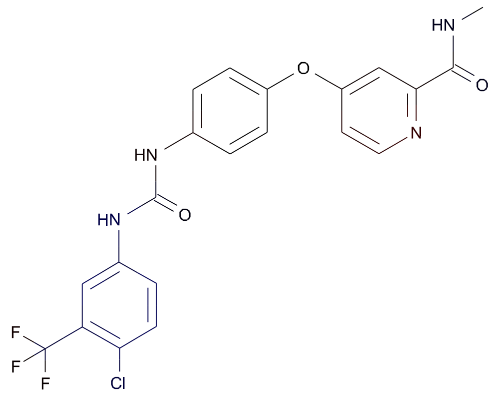

$C_{21}H_{16}ClF_3N_4O_3$
Molecular Weight: 464.82494
ALogP: 4.175
Rotatable Bonds: 6
Acceptors: 4
Donors: 3

Structural Similar Compounds

Name	4;4'-DIAMINO-1;1'-DIANTHRIMIDE	5-NORBORNENE-2;3-DICARBOXYLIC ACID; 1;4;5;6;7;7-HEXACHLORO-	METHANE;TRIS(4-AMINOPHENYL)-
Structure	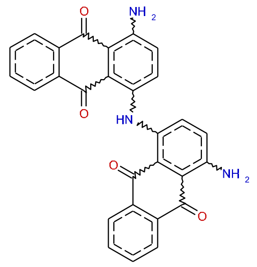	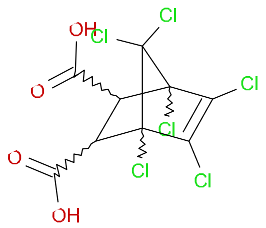	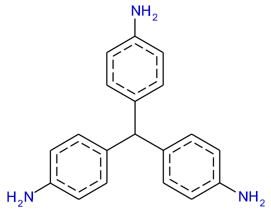
Actual Endpoint	Mild	Moderate_Severe	Moderate_Severe
Predicted Endpoint	Mild	Moderate_Severe	Moderate_Severe
Distance	0.799	0.816	0.827
Reference	28ZPAK-;125;72	28ZPAK-;92;72	28ZPAK-;73;72

Model Prediction

Prediction: Mild
Probability: 0.776
Enrichment: 1.13
Bayesian Score: -1.8
Mahalanobis Distance: 8.95
Mahalanobis Distance p-value: 0.537

Prediction: Positive if the Bayesian score is above the estimated best cutoff value from minimizing the false positive and false negative rate.
Probability: The esimated probability that the sample is in the positive category. This assumes that the Bayesian score follows a normal distribution and is different from the prediction using a cutoff.
Enrichment: An estimate of enrichment, that is, the increased likelihood (versus random) of this sample being in the category.
Bayesian Score: The standard Laplacian-modified Bayesian score.
Mahalanobis Distance: The Mahalanobis distance (MD) is the distance to the center of the training data. The larger the MD, the less trustworthy the prediction.
Mahalanobis Distance p-value: The p-value gives the fraction of training data with an MD greater than or equal to the one for the given sample, assuming normally distributed data. The smaller the p-value, the less trustworthy the prediciton. For highly non-normal X properties (e.g., fingerprints), the MD p-value is wildly inaccurate.

Model Applicability

Unknown features are fingerprint features in the query molecule, but not found or appearing too infrequently in the training set.

1. All properties and OPS components are within expected ranges.

Feature Contribution

Top features for positive contribution

Fingerprint	Bit/Smiles	Feature Structure	Score	Moderate_Severe in training set
FCFP_10	-1695756380	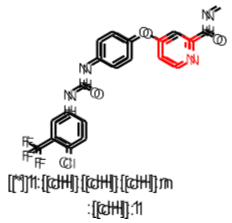	0.285	10 out of 11

FCFP_10	-124655670	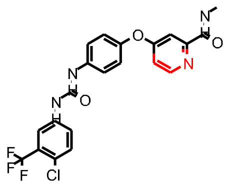 [*]:[cH]:[cH]:n:[*]	0.259	14 out of 16
FCFP_10	-885550502	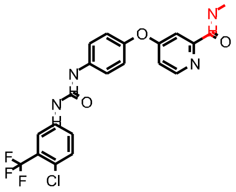 [*]C(=[*])NC	0.239	54 out of 64
**Top Features for negative contribution**				
**Fingerprint**	**Bit/Smiles**	**Feature Structure**	**Score**	**Moderate_Severe in training set**
FCFP_10	2104062943	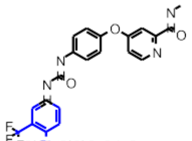 [*]C([*])([*])[c]1:[cH]:[*]:[cH]:[cH]:[c]:1Cl	-0.745	7 out of 24
FCFP_10	-174293376	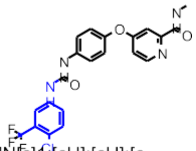 [*]N[c]1:[cH]:[cH]:[c](Cl):[c](:[cH]:1)C([*])([*])[*]	-0.507	0 out of 1
FCFP_10	-1549103449	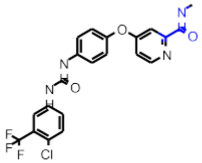 [*]NC(=O)[c](:[*]):[*]	-0.504	2 out of 6

Compound 10

TOPKAT_Ocular_Irritancy_None_vs_Irritant

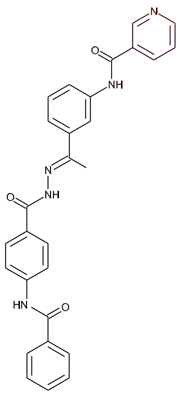

$C_{28}H_{23}N_5O_3$

Molecular Weight: 477.51391

ALogP: 3.082

Rotatable Bonds: 7

Acceptors: 5

Donors: 3

Model Prediction

Prediction: Irritant

Probability: 1

Enrichment: 1.18

Bayesian Score: 1.85

Mahalanobis Distance: 7.1

Mahalanobis Distance p-value: 0.997

Prediction: Positive if the Bayesian score is above the estimated best cutoff value from minimizing the false positive and false negative rate.
Probability: The esimated probability that the sample is in the positive category. This assumes that the Bayesian score follows a normal distribution and is different from the prediction using a cutoff.
Enrichment: An estimate of enrichment, that is, the increased likelihood (versus random) of this sample being in the category.
Bayesian Score: The standard Laplacian-modified Bayesian score.
Mahalanobis Distance: The Mahalanobis distance (MD) is the distance to the center of the training data. The larger the MD, the less trustworthy the prediction.
Mahalanobis Distance p-value: The p-value gives the fraction of training data with an MD greater than or equal to the one for the given sample, assuming normally distributed data. The smaller the p-value, the less trustworthy the prediciton. For highly non-normal X properties (e.g., fingerprints), the MD p-value is wildly inaccurate.

Structural Similar Compounds

Name	4;4'-DIAMINO-1;1'-DIANTHRIMIDE	ANTHRAQUINONE; 1-((2-HYDROXYETHYL)AMINO)-4-(METHYLAMINO)-	1-AMINO-4-BENZOYLAMINO-ANTHRAQUINONE
Structure	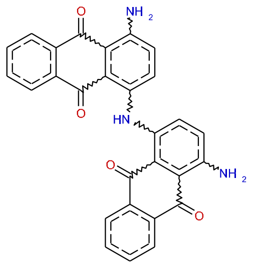	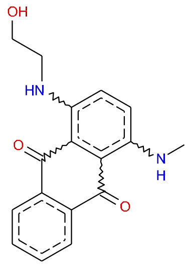	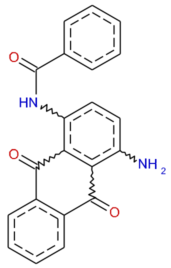
Actual Endpoint	Irritant	Irritant	Irritant
Predicted Endpoint	Irritant	Irritant	Irritant
Distance	0.717	0.781	0.783
Reference	28ZPAK-;125;72	28ZPAK 245;72	28ZPAK-;124;72

Model Applicability

Unknown features are fingerprint features in the query molecule, but not found or appearing too infrequently in the training set.

1. All properties and OPS components are within expected ranges.
2. Unknown FCFP_2 feature: 581019816: [*]NN=C([*])[*]

Feature Contribution

Top features for positive contribution

Fingerprint	Bit/Smiles	Feature Structure	Score	Irritant in training set
FCFP_12	1747237384	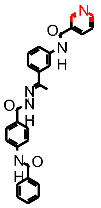 [*]:[cH]:n:[cH]:[*]	0.208	44 out of 44

FCFP_12	-124655670	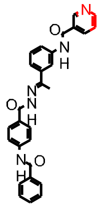 [*]:[cH]:[cH]:n:[*]	0.2	16 out of 16
FCFP_12	-1695756380	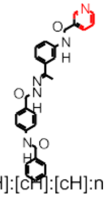 [*]1:[cH]:[cH]:[cH]:n :[cH]:1	0.194	11 out of 11

Top Features for negative contribution

Fingerprint	Bit/Smiles	Feature Structure	Score	Irritant in training set
FCFP_12	-1698724694	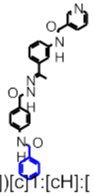 [*]C(=[*])[c]1:[cH]:[cH]:[cH]:[cH]:[cH]:1	-0.0964	107 out of 146
FCFP_12	975909016	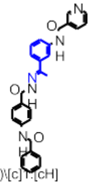 [*]\N=C(/C)\[c]1:[cH] :[cH]:[cH]:[c]([*]): [cH]:1	-0.0639	6 out of 8
FCFP_12	203677720	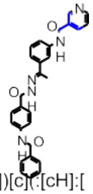 [*]C(=[*])[c](:[cH]:[*]):[cH]:[*]	0	319 out of 382

Sorafenib

TOPKAT_Ocular_Irritancy_None_vs_Irritant

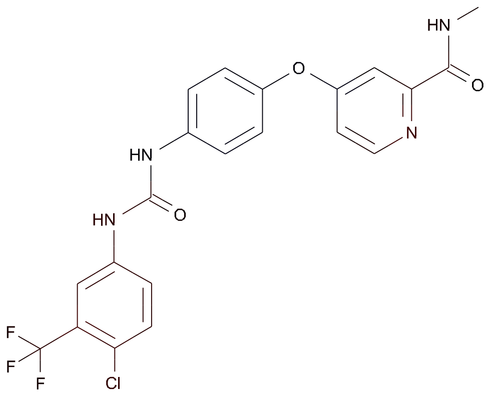

$C_{21}H_{16}ClF_3N_4O_3$
Molecular Weight: 464.82494
ALogP: 4.175
Rotatable Bonds: 6
Acceptors: 4
Donors: 3

Model Prediction

Prediction: Irritant

Probability: 1

Enrichment: 1.18

Bayesian Score: 3.04

Mahalanobis Distance: 6.28

Mahalanobis Distance p-value: 1

Prediction: Positive if the Bayesian score is above the estimated best cutoff value from minimizing the false positive and false negative rate.
Probability: The esimated probability that the sample is in the positive category. This assumes that the Bayesian score follows a normal distribution and is different from the prediction using a cutoff.
Enrichment: An estimate of enrichment, that is, the increased likelihood (versus random) of this sample being in the category.
Bayesian Score: The standard Laplacian-modified Bayesian score.
Mahalanobis Distance: The Mahalanobis distance (MD) is the distance to the center of the training data. The larger the MD, the less trustworthy the prediction.
Mahalanobis Distance p-value: The p-value gives the fraction of training data with an MD greater than or equal to the one for the given sample, assuming normally distributed data. The smaller the p-value, the less trustworthy the prediciton. For highly non-normal X properties (e.g., fingerprints), the MD p-value is wildly inaccurate.

Structural Similar Compounds

Name	BENZANILIDE;2';2'''-DITHIOBIS-	4;4'-DIAMINO-1;1'-DIANTHRIMIDE	5-NORBORNENE-2;3-DICARBOXYLIC ACID; 1;4;5;6;7;7-HEXACHLORO-
Structure	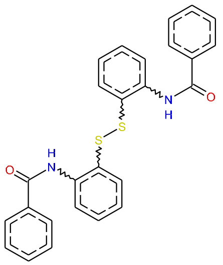	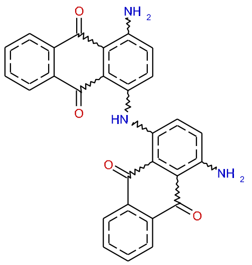	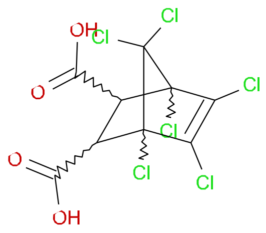
Actual Endpoint	Non-Irritant	Irritant	Irritant
Predicted Endpoint	Non-Irritant	Irritant	Irritant
Distance	0.743	0.791	0.801
Reference	28ZPAK-;173;72	28ZPAK-;125;72	28ZPAK-;92;72

Model Applicability

Unknown features are fingerprint features in the query molecule, but not found or appearing too infrequently in the training set.

1. All properties and OPS components are within expected ranges.

Feature Contribution

Top features for positive contribution				
**Fingerprint**	**Bit/Smiles**	**Feature Structure**	**Score**	**Irritant in training set**
FCFP_12	1747237384	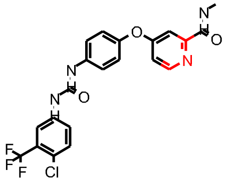 [*]:[cH]:n:[cH]:[*]	0.208	44 out of 44

FCFP_12	-124655670	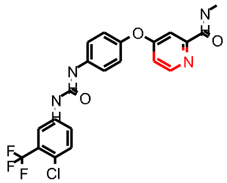 [*]:[cH]:[cH]:n:[*]	0.2	16 out of 16
FCFP_12	-1539132615	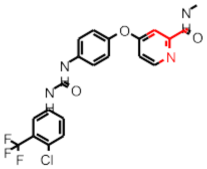 [*]C(=[*])[c](:[cH]:[*]):n:[*]	0.197	13 out of 13

Top Features for negative contribution

Fingerprint	Bit/Smiles	Feature Structure	Score	Irritant in training set
FCFP_12	-747629521	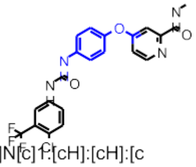 [*]N[c]1:[cH]:[cH]:[c](O[c](:[*]):[*]):[cH]:[cH]:1	-0.268	1 out of 2
FCFP_12	702861189	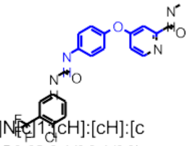 [*]N[c]1:[cH]:[cH]:[c](O[c]2:[cH]:[cH]:[*]:[c]([*]):[cH]:2):[cH]:[cH]:1	-0.268	1 out of 2
FCFP_12	-1270820019	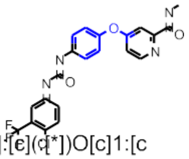 [*]:[c](:[*])O[c]1:[cH]:[cH]:[*]:[cH]:[cH]:1	0	7 out of 9

Compound 10

TOPKAT_Rat_Female_FDA_None_vs_Carcinogen

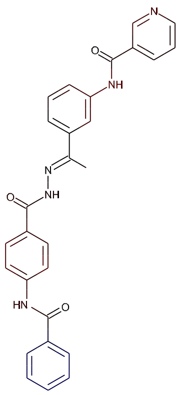

$C_{28}H_{23}N_5O_3$
Molecular Weight: 477.51391
ALogP: 3.082
Rotatable Bonds: 7
Acceptors: 5
Donors: 3

Structural Similar Compounds

Name	Glimepride	Glyburide	Bicalutamide
Structure	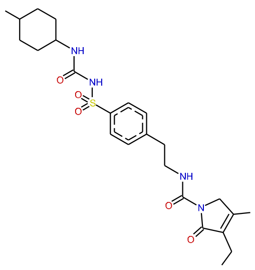	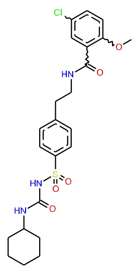	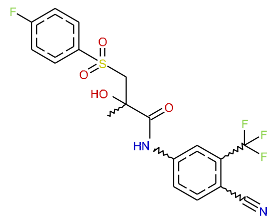
Actual Endpoint	Non-Carcinogen	Non-Carcinogen	Carcinogen
Predicted Endpoint	Non-Carcinogen	Non-Carcinogen	Carcinogen
Distance	0.563	0.601	0.653
Reference	US FDA (Centre for Drug Eval.& Res./Off. Testing & Res.) Sept. 1997	US FDA (Centre for Drug Eval.& Res./Off. Testing & Res.) Sept. 1997	US FDA (Centre for Drug Eval.& Res./Off. Testing & Res.) Sept. 1997

Model Prediction

Prediction: Carcinogen
Probability: 0.303
Enrichment: 0.942
Bayesian Score: 0.237
Mahalanobis Distance: 14.5
Mahalanobis Distance p-value: 2.44e-007

Prediction: Positive if the Bayesian score is above the estimated best cutoff value from minimizing the false positive and false negative rate.
Probability: The esimated probability that the sample is in the positive category. This assumes that the Bayesian score follows a normal distribution and is different from the prediction using a cutoff.
Enrichment: An estimate of enrichment, that is, the increased likelihood (versus random) of this sample being in the category.
Bayesian Score: The standard Laplacian-modified Bayesian score.
Mahalanobis Distance: The Mahalanobis distance (MD) is the distance to the center of the training data. The larger the MD, the less trustworthy the prediction.
Mahalanobis Distance p-value: The p-value gives the fraction of training data with an MD greater than or equal to the one for the given sample, assuming normally distributed data. The smaller the p-value, the less trustworthy the prediciton. For highly non-normal X properties (e.g., fingerprints), the MD p-value is wildly inaccurate.

Model Applicability

Unknown features are fingerprint features in the query molecule, but not found or appearing too infrequently in the training set.

1. All properties and OPS components are within expected ranges.
2. Unknown ECFP_2 feature: 128986386: [*]\N=C(/C)\[c](:[*]):[*]

Feature Contribution

Top features for positive contribution

Fingerprint	Bit/Smiles	Feature Structure	Score	Carcinogen in training set
ECFP_12	-223149939	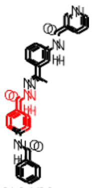 [illegible]	0.613	2 out of 2

ECFP_12	-177077903	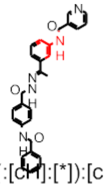 [*]N[c](:[cH]:[*]):[cH]:[*]	0.529	6 out of 10
ECFP_12	-1236483485	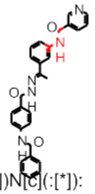 [*]C(=[*])N[c](:[*]):[*]	0.46	9 out of 17
**Top Features for negative contribution**				
**Fingerprint**	**Bit/Smiles**	**Feature Structure**	**Score**	**Carcinogen in training set**
ECFP_12	-281505363	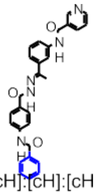 [*][c]1:[cH]:[cH]:[cH]:[cH]:[cH]:1	-0.56	11 out of 64
ECFP_12	1571214559	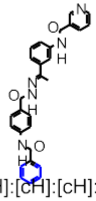 [*]1:[cH]:[cH]:[cH]:[cH]:[cH]:1	-0.56	11 out of 64
ECFP_12	2007300961	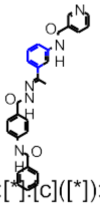 [*][c]1:[*]:[c]([*]):[cH]:[cH]:[cH]:1	-0.426	7 out of 36

Sorafenib

TOPKAT_Rat_Female_FDA_None_vs_Carcinogen

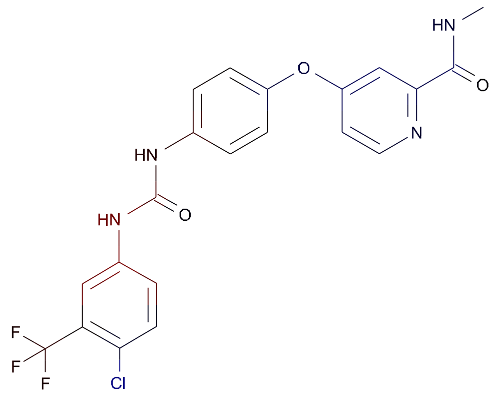

$C_{21}H_{16}ClF_3N_4O_3$
Molecular Weight: 464.82494
ALogP: 4.175
Rotatable Bonds: 6
Acceptors: 4
Donors: 3

Model Prediction

Prediction: Non-Carcinogen

Probability: 0.236

Enrichment: 0.734

Bayesian Score: -3.76

Mahalanobis Distance: 12.2

Mahalanobis Distance p-value: 0.00229

Prediction: Positive if the Bayesian score is above the estimated best cutoff value from minimizing the false positive and false negative rate.
Probability: The esimated probability that the sample is in the positive category. This assumes that the Bayesian score follows a normal distribution and is different from the prediction using a cutoff.
Enrichment: An estimate of enrichment, that is, the increased likelihood (versus random) of this sample being in the category.
Bayesian Score: The standard Laplacian-modified Bayesian score.
Mahalanobis Distance: The Mahalanobis distance (MD) is the distance to the center of the training data. The larger the MD, the less trustworthy the prediction.
Mahalanobis Distance p-value: The p-value gives the fraction of training data with an MD greater than or equal to the one for the given sample, assuming normally distributed data. The smaller the p-value, the less trustworthy the prediciton. For highly non-normal X properties (e.g., fingerprints), the MD p-value is wildly inaccurate.

Structural Similar Compounds

Name	Glimepride	Glyburide	Fluvastatin
Structure	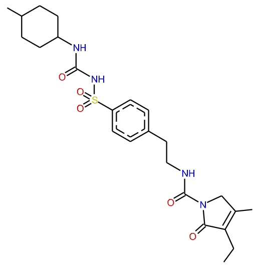	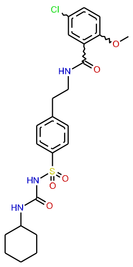	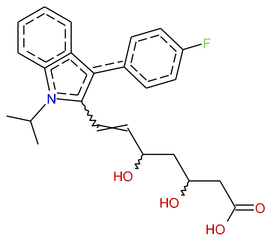
Actual Endpoint	Non-Carcinogen	Non-Carcinogen	Non-Carcinogen
Predicted Endpoint	Non-Carcinogen	Non-Carcinogen	Non-Carcinogen
Distance	0.620	0.635	0.635
Reference	US FDA (Centre for Drug Eval.& Res./Off. Testing & Res.) Sept. 1997	US FDA (Centre for Drug Eval.& Res./Off. Testing & Res.) Sept. 1997	US FDA (Centre for Drug Eval.& Res./Off. Testing & Res.) Sept. 1997

Model Applicability

Unknown features are fingerprint features in the query molecule, but not found or appearing too infrequently in the training set.

1. All properties and OPS components are within expected ranges.

Feature Contribution

Top features for positive contribution

Fingerprint	Bit/Smiles	Feature Structure	Score	Carcinogen in training set
ECFP_12	-970385855	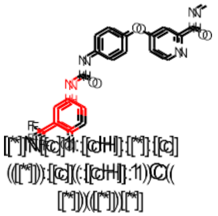	0.613	2 out of 2

ECFP_12	-177077903	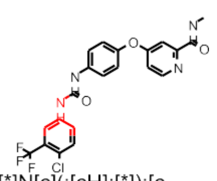 [*]N[c](:[cH]:[*]):[cH]:[*]	0.529	6 out of 10
ECFP_12	-1236483485	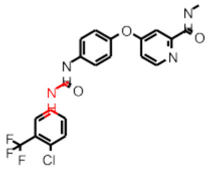 [*]C(=[*])N[c](:[*]):[*]	0.46	9 out of 17

Top Features for negative contribution

Fingerprint	Bit/Smiles	Feature Structure	Score	Carcinogen in training set
ECFP_12	1335691903	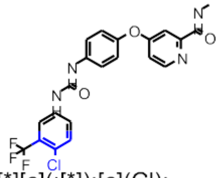 [*][c](:[*]):[c](Cl):[cH]:[*]	-1.11	2 out of 26
ECFP_12	99947387	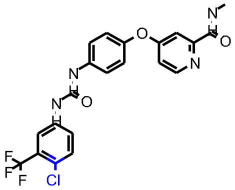 [*]:[c](:[*])Cl	-0.817	8 out of 62
ECFP_12	1413420509	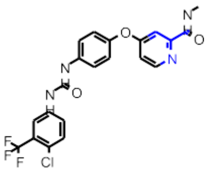 [*]C(=[*])[c](:[cH]:[*]):n:[*]	-0.661	0 out of 3

Compound 10

TOPKAT_Rat_Female_FDA_Single_vs_Multiple

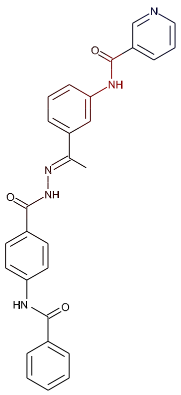

$C_{28}H_{23}N_5O_3$

Molecular Weight: 477.51391

ALogP: 3.082

Rotatable Bonds: 7

Acceptors: 5

Donors: 3

Model Prediction

Prediction: Multiple-Carcinogen

Probability: 0.605

Enrichment: 1.62

Bayesian Score: 3.33

Mahalanobis Distance: 13.1

Mahalanobis Distance p-value: 0.000192

Prediction: Positive if the Bayesian score is above the estimated best cutoff value from minimizing the false positive and false negative rate.
Probability: The esimated probability that the sample is in the positive category. This assumes that the Bayesian score follows a normal distribution and is different from the prediction using a cutoff.
Enrichment: An estimate of enrichment, that is, the increased likelihood (versus random) of this sample being in the category.
Bayesian Score: The standard Laplacian-modified Bayesian score.
Mahalanobis Distance: The Mahalanobis distance (MD) is the distance to the center of the training data. The larger the MD, the less trustworthy the prediction.
Mahalanobis Distance p-value: The p-value gives the fraction of training data with an MD greater than or equal to the one for the given sample, assuming normally distributed data. The smaller the p-value, the less trustworthy the prediciton. For highly non-normal X properties (e.g., fingerprints), the MD p-value is wildly inaccurate.

Structural Similar Compounds

Name	Bicalutamide	Torsemide	Ursodiol
Structure	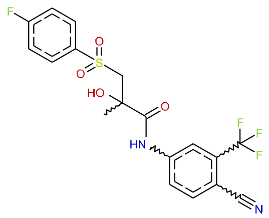	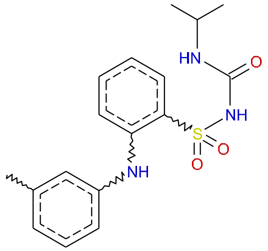	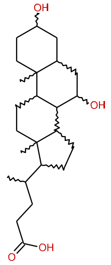
Actual Endpoint	Multiple-Carcinogen	Single-Carcinogen	Single-Carcinogen
Predicted Endpoint	Multiple-Carcinogen	Single-Carcinogen	Single-Carcinogen
Distance	0.639	0.715	0.738
Reference	US FDA (Centre for Drug Eval.& Res./Off. Testing & Res.) Sept. 1997	US FDA (Centre for Drug Eval.& Res./Off. Testing & Res.) Sept. 1997	US FDA (Centre for Drug Eval.& Res./Off. Testing & Res.) Sept. 1997

Model Applicability

Unknown features are fingerprint features in the query molecule, but not found or appearing too infrequently in the training set.

1. All properties and OPS components are within expected ranges.

Feature Contribution

Top features for positive contribution

Fingerprint	Bit/Smiles	Feature Structure	Score	Multiple-Carcinogen in training set
SCFP_4	1631845520	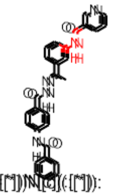 [*]C(=[*])N[c](:[*]):[*]	0.601	6 out of 9

SCFP_4	-1375926917	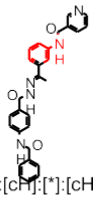 [*]N[c]1:[cH]:[*]:[cH]:[cH]:[cH]:1	0.522	6 out of 10
SCFP_4	1205586762	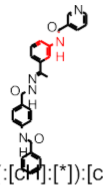 [*]N[c](:[cH]:[*]):[cH]:[*]	0.451	7 out of 13

Top Features for negative contribution				
**Fingerprint**	**Bit/Smiles**	**Feature Structure**	**Score**	**Multiple-Carcinogen in training set**
SCFP_4	1188429584	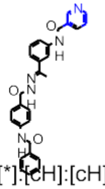 [*][c]1:[*]:[cH]:[cH]:n:[cH]:1	-0.666	0 out of 3
SCFP_4	-758850909	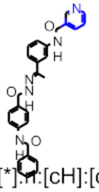 [*][c]1:[*]:n:[cH]:[cH]:[cH]:1	-0.489	0 out of 2
SCFP_4	-937094999	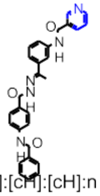 [*]1:[cH]:[cH]:[cH]:n:[cH]:1	-0.368	1 out of 6

Compound 10

TOPKAT_Rat_Male_FDA_None_vs_Carcinogen

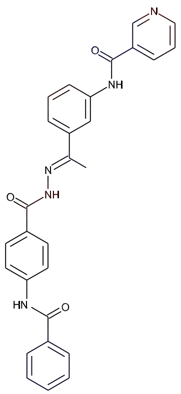

$C_{28}H_{23}N_5O_3$
Molecular Weight: 477.51391
ALogP: 3.082
Rotatable Bonds: 7
Acceptors: 5
Donors: 3

Structural Similar Compounds

Name	Glimepride	Glyburide	Glipizide
Structure	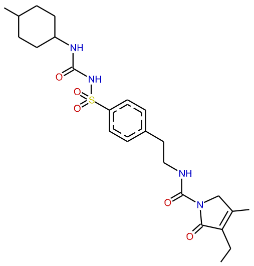	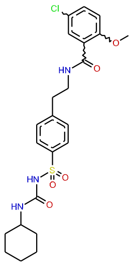	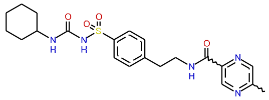
Actual Endpoint	Non-Carcinogen	Non-Carcinogen	Non-Carcinogen
Predicted Endpoint	Non-Carcinogen	Non-Carcinogen	Non-Carcinogen
Distance	0.548	0.587	0.634
Reference	US FDA (Centre for Drug Eval.& Res./Off. Testing & Res.) Sept. 1997	US FDA (Centre for Drug Eval.& Res./Off. Testing & Res.) Sept. 1997	US FDA (Centre for Drug Eval.& Res./Off. Testing & Res.) Sept. 1997

Model Applicability

Unknown features are fingerprint features in the query molecule, but not found or appearing too infrequently in the training set.

1. All properties and OPS components are within expected ranges.

Model Prediction

Prediction: Non-Carcinogen
Probability: 0.335
Enrichment: 1
Bayesian Score: -0.772
Mahalanobis Distance: 14.5
Mahalanobis Distance p-value: 5.99e-006

Prediction: Positive if the Bayesian score is above the estimated best cutoff value from minimizing the false positive and false negative rate.
Probability: The esimated probability that the sample is in the positive category. This assumes that the Bayesian score follows a normal distribution and is different from the prediction using a cutoff.
Enrichment: An estimate of enrichment, that is, the increased likelihood (versus random) of this sample being in the category.
Bayesian Score: The standard Laplacian-modified Bayesian score.
Mahalanobis Distance: The Mahalanobis distance (MD) is the distance to the center of the training data. The larger the MD, the less trustworthy the prediction.
Mahalanobis Distance p-value: The p-value gives the fraction of training data with an MD greater than or equal to the one for the given sample, assuming normally distributed data. The smaller the p-value, the less trustworthy the prediciton. For highly non-normal X properties (e.g., fingerprints), the MD p-value is wildly inaccurate.

Feature Contribution

Top features for positive contribution

Fingerprint	Bit/Smiles	Feature Structure	Score	Carcinogen in training set
SCFP_6	-347048986	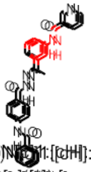 [*]C(=[*])N[illegible]1:[cH]:[cH]:[*]:[c](:[*]):[cH]:1	0.615	5 out of 7

SCFP_6	698322229	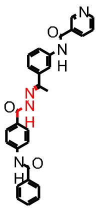 [*]C(=[*])NN=[*]	0.415	1 out of 1
SCFP_6	-105808146	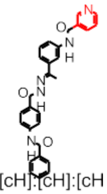 [*][c]1:[cH]:[cH]:[cH]:n:[cH]:1	0.415	1 out of 1

Top Features for negative contribution

Fingerprint	Bit/Smiles	Feature Structure	Score	Carcinogen in training set
SCFP_6	1653911926	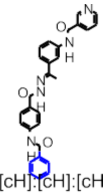 [*][c]1:[cH]:[cH]:[cH]:[cH]:[cH]:1	-0.504	12 out of 64
SCFP_6	1257084377	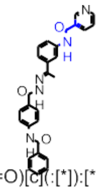 [*]NC(=O)[c](:[*]):[*]	-0.436	4 out of 21
SCFP_6	-331724199	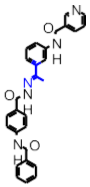 [*]\N=C(/C)\[c](:[*]):[*]	-0.278	0 out of 1

Sorafenib

TOPKAT_Rat_Male_FDA_None_vs_Carcinogen

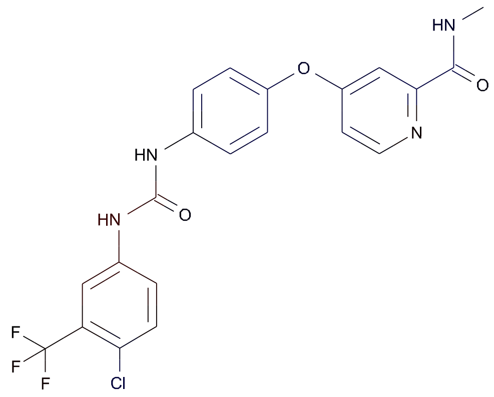

$C_{21}H_{16}ClF_3N_4O_3$
Molecular Weight: 464.82494

ALogP: 4.175

Rotatable Bonds: 6

Acceptors: 4

Donors: 3

Structural Similar Compounds

Name	Glyburide	Glimepride	Fluvastatin
Structure	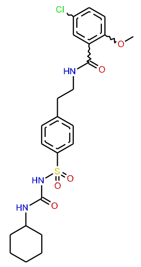	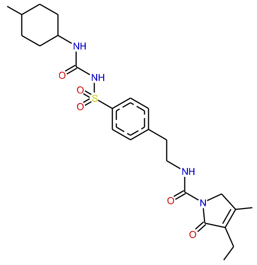	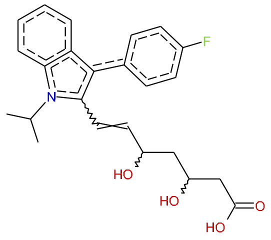
Actual Endpoint	Non-Carcinogen	Non-Carcinogen	Carcinogen
Predicted Endpoint	Non-Carcinogen	Non-Carcinogen	Carcinogen
Distance	0.593	0.600	0.615
Reference	US FDA (Centre for Drug Eval.& Res./Off. Testing & Res.) Sept. 1997	US FDA (Centre for Drug Eval.& Res./Off. Testing & Res.) Sept. 1997	US FDA (Centre for Drug Eval.& Res./Off. Testing & Res.) Sept. 1997

Model Applicability

Unknown features are fingerprint features in the query molecule, but not found or appearing too infrequently in the training set.

1. All properties and OPS components are within expected ranges.

Model Prediction

Prediction: Non-Carcinogen

Probability: 0.293

Enrichment: 0.878

Bayesian Score: -2.4

Mahalanobis Distance: 17.6

Mahalanobis Distance p-value: 1.1e-012

Prediction: Positive if the Bayesian score is above the estimated best cutoff value from minimizing the false positive and false negative rate.
Probability: The esimated probability that the sample is in the positive category. This assumes that the Bayesian score follows a normal distribution and is different from the prediction using a cutoff.
Enrichment: An estimate of enrichment, that is, the increased likelihood (versus random) of this sample being in the category.
Bayesian Score: The standard Laplacian-modified Bayesian score.
Mahalanobis Distance: The Mahalanobis distance (MD) is the distance to the center of the training data. The larger the MD, the less trustworthy the prediction.
Mahalanobis Distance p-value: The p-value gives the fraction of training data with an MD greater than or equal to the one for the given sample, assuming normally distributed data. The smaller the p-value, the less trustworthy the prediciton. For highly non-normal X properties (e.g., fingerprints), the MD p-value is wildly inaccurate.

Feature Contribution

Top features for positive contribution

Fingerprint	Bit/Smiles	Feature Structure	Score	Carcinogen in training set
SCFP_6	-347048986	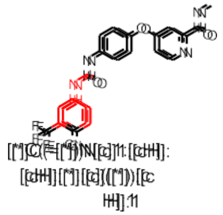	0.615	5 out of 7

SCFP_6	-754059116	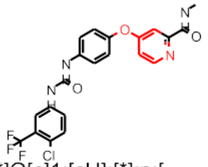 [*]O[c]1:[cH]:[*]:n:[cH]:[cH]:1	0.415	1 out of 1
SCFP_6	-531283893	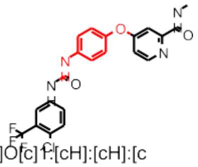 [*]O[c]1:[cH]:[cH]:[c](NC(=[*])[*]):[cH]:[cH]:1	0.273	2 out of 4
**Top Features for negative contribution**				
**Fingerprint**	**Bit/Smiles**	**Feature Structure**	**Score**	**Carcinogen in training set**
SCFP_6	-827073191	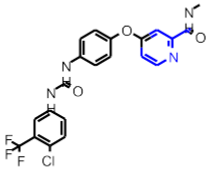 [*]C(=[*])[c]1:[cH]:[*]:[cH]:[cH]:n:1	-0.674	0 out of 3
SCFP_6	-488587948	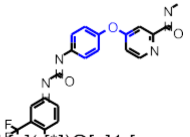 [*]:[c](:[*])O[c]1:[cH]:[cH]:[*]:[cH]:[cH]:1	-0.496	0 out of 2
SCFP_6	-975241316	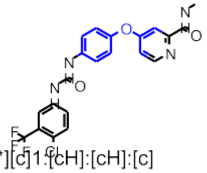 [*][c]1:[cH]:[cH]:[c](O[c](:[cH]:[*]):[cH]:[*]):[cH]:[cH]:1	-0.496	0 out of 2

Compound 10

TOPKAT_Skin_Irritancy_None_vs_Irritant

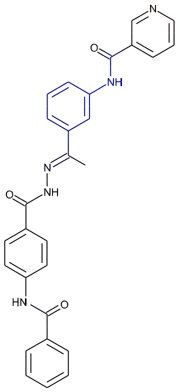

$C_{28}H_{23}N_5O_3$

Molecular Weight: 477.51391

ALogP: 3.082

Rotatable Bonds: 7

Acceptors: 5

Donors: 3

Structural Similar Compounds

Name	Benzenesulfonic acid, 2,2'-(4,4'-biphenylylenedivinylene)di-, disod ium salt	2-Anthracenesulfonic acid, 1-amino-9,10-dihydro-9,10-dioxo-4-(2,4,6 -trimethylanilino)-, monosodium salt	5-Norbornene-2,3-dicarboxylic acid, 1,4,5,6,7,7-hexachloro-
Structure	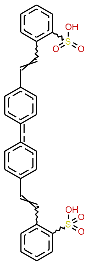	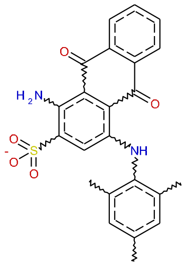	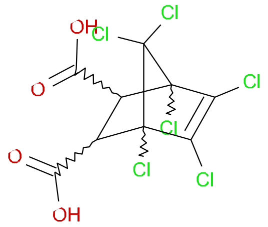
Actual Endpoint	Irritant	Irritant	Irritant
Predicted Endpoint	Non-Irritant	Non-Irritant	Irritant
Distance	0.834	0.862	0.910
Reference	MVCRB3 MVC-Report. (Stockholm, Sweden) No.1-2, 1972-73. Discontinued. Volu me(issue)/page/year: 2,193,1973	85JCAE "Prehled Prumyslove Toxikologie; Organicke Latky," Marhold, J., Prague , Czechoslovakia, Avicenum, 1986 Volume(issue)/page/year: -,1327,1986	85JCAE "Prehled Prumyslove Toxikologie; Organicke Latky," Marhold, J., Prague , Czechoslovakia, Avicenum, 1986 Volume(issue)/page/year: -,581,1986

Model Prediction

Prediction: Non-Irritant

Probability: 0.684

Enrichment: 0.743

Bayesian Score: -4

Mahalanobis Distance: 7.39

Mahalanobis Distance p-value: 0.981

Prediction: Positive if the Bayesian score is above the estimated best cutoff value from minimizing the false positive and false negative rate.
Probability: The esimated probability that the sample is in the positive category. This assumes that the Bayesian score follows a normal distribution and is different from the prediction using a cutoff.
Enrichment: An estimate of enrichment, that is, the increased likelihood (versus random) of this sample being in the category.
Bayesian Score: The standard Laplacian-modified Bayesian score.
Mahalanobis Distance: The Mahalanobis distance (MD) is the distance to the center of the training data. The larger the MD, the less trustworthy the prediction.
Mahalanobis Distance p-value: The p-value gives the fraction of training data with an MD greater than or equal to the one for the given sample, assuming normally distributed data. The smaller the p-value, the less trustworthy the prediciton. For highly non-normal X properties (e.g., fingerprints), the MD p-value is wildly inaccurate.

Model Applicability

Unknown features are fingerprint features in the query molecule, but not found or appearing too infrequently in the training set.

1. All properties and OPS components are within expected ranges.

Feature Contribution

Top features for positive contribution				
**Fingerprint**	**Bit/Smiles**	**Feature Structure**	**Score**	**Irritant in training set**

FCFP_12	-124655670	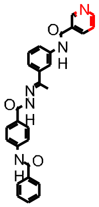 [*]:[cH]:[cH]:n:[*]	0.0821	13 out of 13
FCFP_12	-1695756380	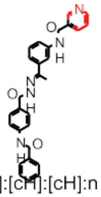 [*]1:[cH]:[cH]:[cH]:n:[cH]:1	0.0772	7 out of 7
FCFP_12	730557100	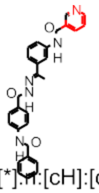 [*][c]1:[*]:n:[cH]:[cH]:[cH]:1	0.0756	6 out of 6
**Top Features for negative contribution**				
**Fingerprint**	**Bit/Smiles**	**Feature Structure**	**Score**	**Irritant in training set**
FCFP_12	-1838187238	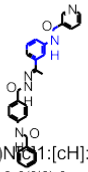 [*]C(=[*])N[c]1:[cH]:[cH]:[*]:[c]([*]):[cH]:1	-0.692	5 out of 12
FCFP_12	-792685140	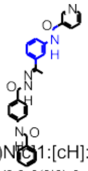 [*]C(=[*])N[c]1:[cH]:[cH]:[cH]:[c]([*]):[cH]:1	-0.65	0 out of 1

FCFP_12	1294255210	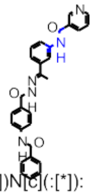 [*]C(=[*])N[c](:[*]):[*]	-	12 out of 22

Sorafenib

TOPKAT_Skin_Irritancy_None_vs_Irritant

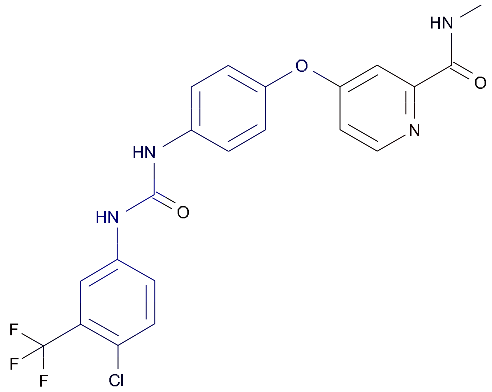

$C_{21}H_{16}ClF_3N_4O_3$
Molecular Weight: 464.82494

ALogP: 4.175

Rotatable Bonds: 6

Acceptors: 4

Donors: 3

Structural Similar Compounds

Name	5-Norbornene-2,3-dicarboxylic acid, 1,4,5,6,7,7-hexachloro-	Benzenesulfonic acid, 2,2'-(4,4'-biphenylylenedivinylene)di-, disod ium salt	Sulfide, bis(4-t-butyl-m-cresyl)-
Structure	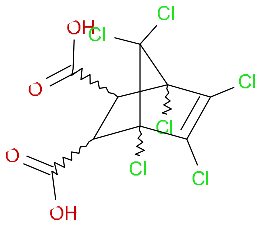	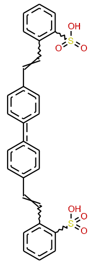	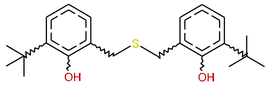
Actual Endpoint	Irritant	Irritant	Irritant
Predicted Endpoint	Irritant	Non-Irritant	Irritant
Distance	0.844	0.871	0.884
Reference	85JCAE "Prehled Prumyslove Toxikologie; Organicke Latky," Marhold, J., Prague , Czechoslovakia, Avicenum, 1986 Volume(issue)/page/year: -,581,1986	MVCRB3 MVC-Report. (Stockholm, Sweden) No.1-2, 1972-73. Discontinued. Volu me(issue)/page/year: 2,193,1973	AMIHBC AMA Archives of Industrial Hygiene and Occupational Medicine. (Chicago , IL) V.2-10, 1950-54. For publisher information, see AEHLAU. Volume(issue)/pag e/year: 5,311,1952

Model Prediction

Prediction: Non-Irritant

Probability: 0.264

Enrichment: 0.287

Bayesian Score: -5.23

Mahalanobis Distance: 8.27

Mahalanobis Distance p-value: 0.791

Prediction: Positive if the Bayesian score is above the estimated best cutoff value from minimizing the false positive and false negative rate.
Probability: The esimated probability that the sample is in the positive category. This assumes that the Bayesian score follows a normal distribution and is different from the prediction using a cutoff.
Enrichment: An estimate of enrichment, that is, the increased likelihood (versus random) of this sample being in the category.
Bayesian Score: The standard Laplacian-modified Bayesian score.
Mahalanobis Distance: The Mahalanobis distance (MD) is the distance to the center of the training data. The larger the MD, the less trustworthy the prediction.
Mahalanobis Distance p-value: The p-value gives the fraction of training data with an MD greater than or equal to the one for the given sample, assuming normally distributed data. The smaller the p-value, the less trustworthy the prediciton. For highly non-normal X properties (e.g., fingerprints), the MD p-value is wildly inaccurate.

Model Applicability

Unknown features are fingerprint features in the query molecule, but not found or appearing too infrequently in the training set.

1. All properties and OPS components are within expected ranges.

Feature Contribution

Top features for positive contribution

Fingerprint	Bit/Smiles	Feature Structure	Score	Irritant in training set

FCFP_12	-124655670	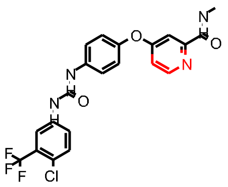 [*]:[cH]:[cH]:n:[*]	0.0821	13 out of 13
FCFP_12	-1539132615	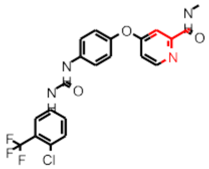 [*]C(=[*])[c](:[cH]:[*]):n:[*]	0.0795	9 out of 9
FCFP_12	-1695756380	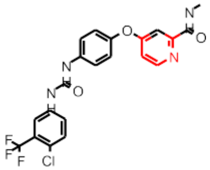 [*]1:[cH]:[cH]:[cH]:n:[cH]:1	0.0772	7 out of 7
**Top Features for negative contribution**				
**Fingerprint**	**Bit/Smiles**	**Feature Structure**	**Score**	**Irritant in training set**
FCFP_12	-789307649	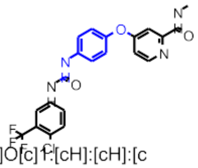 [*]O[c]1:[cH]:[cH]:[c](NC(=[*])[*]):[cH]:[cH]:1	-1.54	0 out of 4
FCFP_12	-1838187238	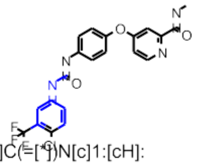 [*]C(=[*])N[c]1:[cH]:[cH]:[*]:[c]([*]):[cH]:1	-0.692	5 out of 12

FCFP_12	1294255210	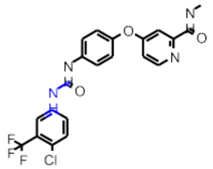 [*]C(=[*])N[c](:[*]):[*]	-	12 out of 22

Compound 10

TOPKAT_Carcinogenic_Potency_TD50_Mouse

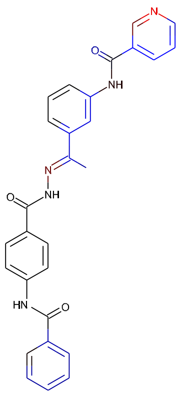

$C_{28}H_{23}N_5O_3$

Molecular Weight: 477.51391

ALogP: 3.082

Rotatable Bonds: 7

Acceptors: 5

Donors: 3

Structural Similar Compounds

Name	Ochratoxin A	542	4-Chloro-6-(2,3-xylidino)-2-pyri•mi•dinylthio(N-b-hydroxy•ethyl) acetamide
Structure	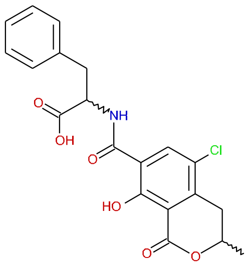	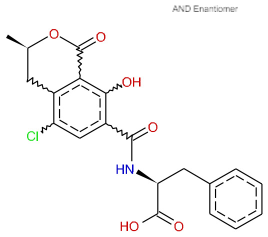	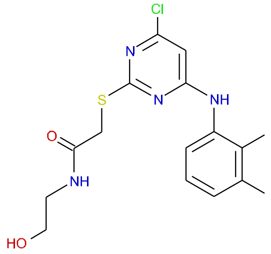
Actual Endpoint (-log C)	4.79932	4.79932	3.91517
Predicted Endpoint (-log C)	3.6353	3.6353	3.92186
Distance	0.816	0.816	0.869
Reference	CPDB	CPDB	CPDB

Model Prediction

Prediction: 22.2

Unit: mg/kg_body_weight/day

Mahalanobis Distance: 12

Mahalanobis Distance p-value: 1.83e-005

Mahalanobis Distance: The Mahalanobis distance (MD) is a generalization of the Euclidean distance that accounts for correlations among the X properties. It is calculated as the distance to the center of the training data. The larger the MD, the less trustworthy the prediction.
Mahalanobis Distance p-value: The p-value gives the fraction of training data with an MD greater than or equal to the one for the given sample, assuming normally distributed data. The smaller the p-value, the less trustworthy the prediciton. For highly non-normal X properties (e.g., fingerprints), the MD p-value is wildly inaccurate.

Model Applicability

Unknown features are fingerprint features in the query molecule, but not found or appearing too infrequently in the training set.

1. All properties and OPS components are within expected ranges.
2. Unknown ECFP_2 feature: 128986386: [*]\N=C(/C)\[c](:[*]):[*]
3. Unknown ECFP_2 feature: 560380707: [*]NN=C([*])[*]

Feature Contribution

Top features for positive contribution

Fingerprint	Bit/Smiles	Feature Structure	Score
ECFP_6	655739385	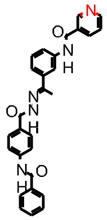 [*]:n:[*]	0.229

ECFP_6	-175146122	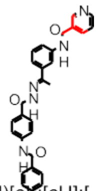 [*]C(=[*])[c](:[cH]:[*]):[cH]:[*]	0.107
ECFP_6	-1087070950	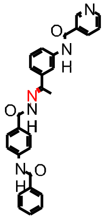 [*]N=[*]	0.104

Top Features for negative contribution

Fingerprint	Bit/Smiles	Feature Structure	Score
ECFP_6	1996767644	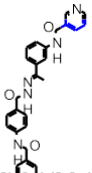 [*][c](:[*]):[cH]:[cH]:[*]	-0.251
ECFP_6	642810091	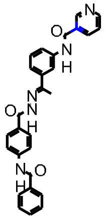 [*][c](:[*]):[*]	-0.247
ECFP_6	-182236392	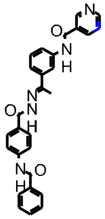 [*]:[cH]:[*]	-0.232

Sorafenib

TOPKAT_Carcinogenic_Potency_TD50_Mouse

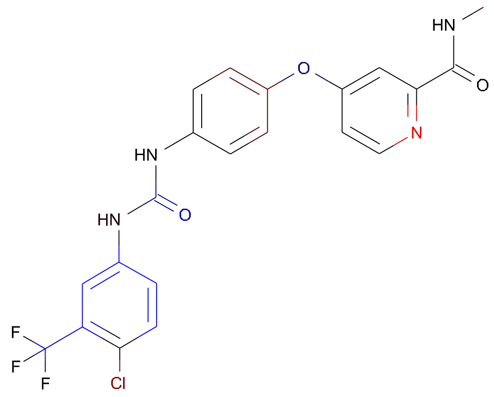

$C_{21}H_{16}ClF_3N_4O_3$
Molecular Weight: 464.82494

ALogP: 4.175

Rotatable Bonds: 6

Acceptors: 4

Donors: 3

Structural Similar Compounds

Name	Ochratoxin A	542	4-Chloro-6-(2,3-xylidino)-2-pyri•mi•dinylthio(N-b-hydroxy•ethyl) acetamide
Structure	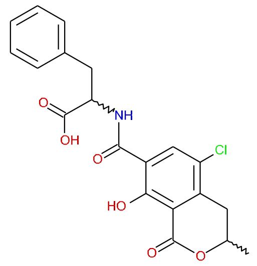	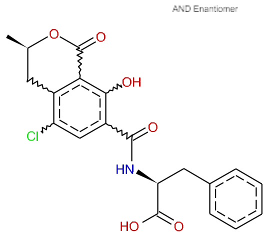	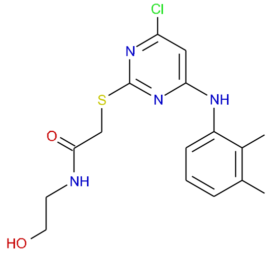
Actual Endpoint (-log C)	4.79932	4.79932	3.91517
Predicted Endpoint (-log C)	3.6353	3.6353	3.92186
Distance	0.718	0.718	0.738
Reference	CPDB	CPDB	CPDB

Model Prediction

Prediction: 19.2

Unit: mg/kg_body_weight/day

Mahalanobis Distance: 12.4

Mahalanobis Distance p-value: 2.94e-006

Mahalanobis Distance: The Mahalanobis distance (MD) is a generalization of the Euclidean distance that accounts for correlations among the X properties. It is calculated as the distance to the center of the training data. The larger the MD, the less trustworthy the prediction.
Mahalanobis Distance p-value: The p-value gives the fraction of training data with an MD greater than or equal to the one for the given sample, assuming normally distributed data. The smaller the p-value, the less trustworthy the prediciton. For highly non-normal X properties (e.g., fingerprints), the MD p-value is wildly inaccurate.

Model Applicability

Unknown features are fingerprint features in the query molecule, but not found or appearing too infrequently in the training set.

1. All properties and OPS components are within expected ranges.
2. Unknown ECFP_2 feature: 1338334141: [*]C(=[*])NC
3. Unknown ECFP_2 feature: 1413420509: [*]C(=[*])[c](:n:[*]):c:[*]

Feature Contribution

Top features for positive contribution			
**Fingerprint**	**Bit/Smiles**	**Feature Structure**	**Score**
ECFP_6	655739385	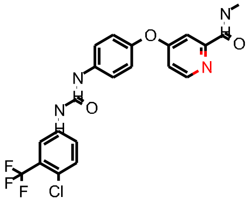 [*]:n:[*]	0.229

ECFP_6	-817402818	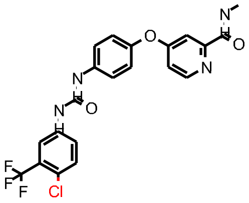 [*]Cl	0.129
ECFP_6	-176455838	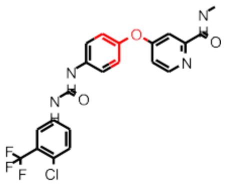 [*]O[c](:[cH]:[*]):[cH]:[*]	0.0818

Top Features for negative contribution

Fingerprint	Bit/Smiles	Feature Structure	Score
ECFP_6	1996767644	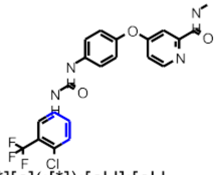 [*][c](:[*]):[cH]:[cH]:[*]	-0.251
ECFP_6	642810091	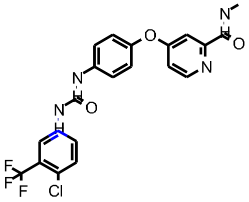 [*][c](:[*]):[*]	-0.247
ECFP_6	-182236392	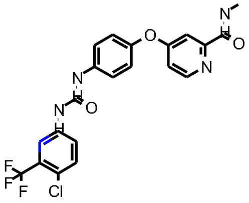 [*]:[cH]:[*]	-0.232

Compound 10

TOPKAT_Carcinogenic_Potency_TD50_Rat

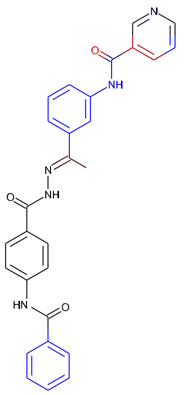

$C_{28}H_{23}N_5O_3$

Molecular Weight: 477.51391

ALogP: 3.082

Rotatable Bonds: 7

Acceptors: 5

Donors: 3

Model Prediction

Prediction: 45.4

Unit: mg/kg_body_weight/day

Mahalanobis Distance: 13

Mahalanobis Distance p-value: 5.88e-006

Mahalanobis Distance: The Mahalanobis distance (MD) is a generalization of the Euclidean distance that accounts for correlations among the X properties. It is calculated as the distance to the center of the training data. The larger the MD, the less trustworthy the prediction.

Mahalanobis Distance p-value: The p-value gives the fraction of training data with an MD greater than or equal to the one for the given sample, assuming normally distributed data. The smaller the p-value, the less trustworthy the prediciton. For highly non-normal X properties (e.g., fingerprints), the MD p-value is wildly inaccurate.

Structural Similar Compounds

Name	913	Fluvastatin	Ochratoxin A
Structure	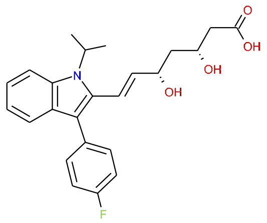	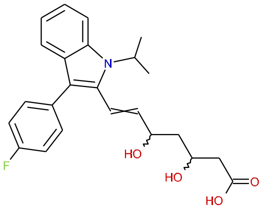	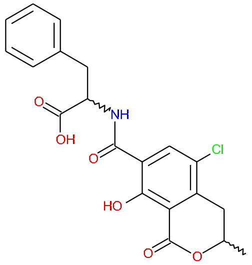
Actual Endpoint (-log C)	3.51742	3.51742	6.47264
Predicted Endpoint (-log C)	5.41573	5.41573	5.06501
Distance	0.732	0.732	0.770
Reference	CPDB	CPDB	CPDB

Model Applicability

Unknown features are fingerprint features in the query molecule, but not found or appearing too infrequently in the training set.

1. All properties and OPS components are within expected ranges.

Feature Contribution

Top features for positive contribution

Fingerprint	Bit/Smiles	Feature Structure	Score
FCFP_6	1	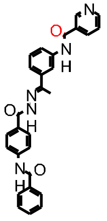 [*]=O	0.234

Fingerprint	Bit/Smiles	Feature Structure	Score
FCFP_6	730557100	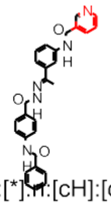 [*][c]1:[*]:n:[cH]:[cH]:[cH]:1	0.141
FCFP_6	203677720	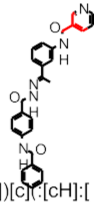 [*]C(=[*])[c](:[cH]:[*]):[cH]:[*]	0.137
**Top Features for negative contribution**			
**Fingerprint**	**Bit/Smiles**	**Feature Structure**	**Score**
FCFP_6	991735244	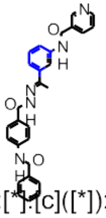 [*][c]1:[*]:[c]([*]):[cH]:[cH]:[cH]:1	-0.422
FCFP_6	-2093839777	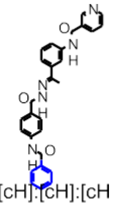 [*][c]1:[cH]:[cH]:[cH]:[cH]:[cH]:1	-0.378
FCFP_6	16	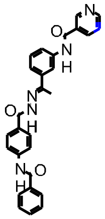 [*]:[cH]:[*]	-0.354

Sorafenib

TOPKAT_Carcinogenic_Potency_TD50_Rat

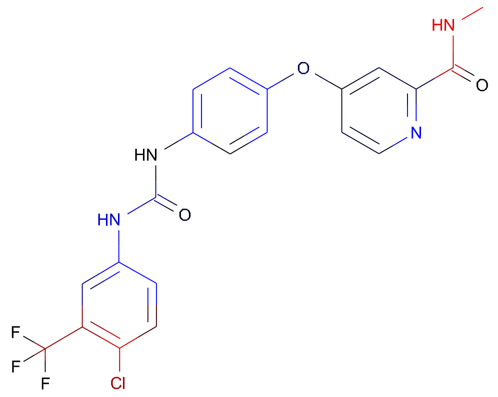

$C_{21}H_{16}ClF_3N_4O_3$
Molecular Weight: 464.82494

ALogP: 4.175

Rotatable Bonds: 6

Acceptors: 4

Donors: 3

Model Prediction

Prediction: 14.2

Unit: mg/kg_body_weight/day

Mahalanobis Distance: 20.4

Mahalanobis Distance p-value: 9.56e-031

Mahalanobis Distance: The Mahalanobis distance (MD) is a generalization of the Euclidean distance that accounts for correlations among the X properties. It is calculated as the distance to the center of the training data. The larger the MD, the less trustworthy the prediction.
Mahalanobis Distance p-value: The p-value gives the fraction of training data with an MD greater than or equal to the one for the given sample, assuming normally distributed data. The smaller the p-value, the less trustworthy the prediciton. For highly non-normal X properties (e.g., fingerprints), the MD p-value is wildly inaccurate.

Structural Similar Compounds

Name	Fluvastatin	913	Ochratoxin A
Structure	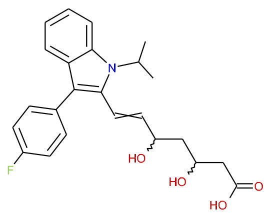	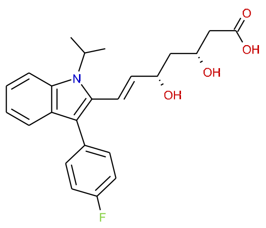	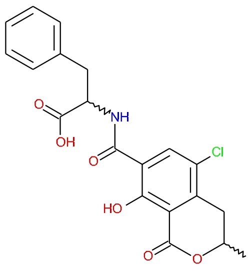
Actual Endpoint (-log C)	3.51742	3.51742	6.47264
Predicted Endpoint (-log C)	5.41573	5.41573	5.06501
Distance	0.597	0.597	0.666
Reference	CPDB	CPDB	CPDB

Model Applicability

Unknown features are fingerprint features in the query molecule, but not found or appearing too infrequently in the training set.

1. All properties and OPS components are within expected ranges.
2. Unknown FCFP_2 feature: -1029533685: [*]:[c](:[*])C(F)(F)F

Feature Contribution

Top features for positive contribution

Fingerprint	Bit/Smiles	Feature Structure	Score
FCFP_6	1	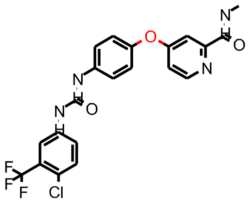 [*]=O	0.234

FCFP_6	-885550502	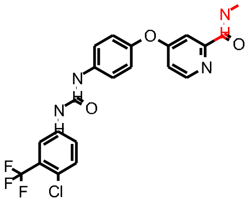 [*]C(=[*])NC	0.229
FCFP_6	32	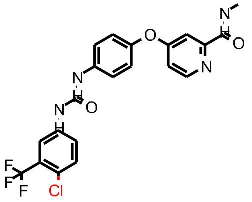 [*]Cl	0.154
**Top Features for negative contribution**			
**Fingerprint**	**Bit/Smiles**	**Feature Structure**	**Score**
FCFP_6	16	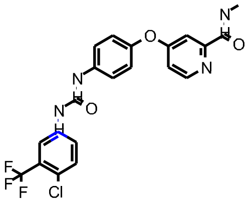 [*]:[cH]:[*]	-0.354
FCFP_6	590925877	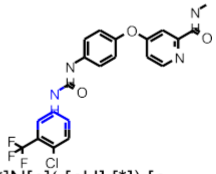 [*]N[c](:[cH]:[*]):[cH]:[*]	-0.323
FCFP_6	1674451008	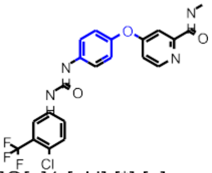 [*]O[c]1:[cH]:[*]:[c]([*]):[cH]:[cH]:1	-0.233

Compound 10

TOPKAT_Chronic_LOAEL

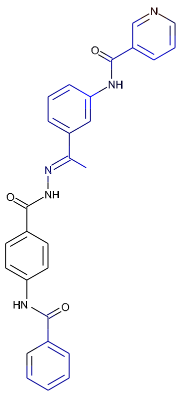

$C_{28}H_{23}N_{5}O_{3}$

Molecular Weight: 477.51391

ALogP: 3.082

Rotatable Bonds: 7

Acceptors: 5

Donors: 3

Model Prediction

Prediction: 0.317

Unit: g/kg_body_weight

Mahalanobis Distance: 31.9

Mahalanobis Distance p-value: 3.77e-028

Mahalanobis Distance: The Mahalanobis distance (MD) is a generalization of the Euclidean distance that accounts for correlations among the X properties. It is calculated as the distance to the center of the training data. The larger the MD, the less trustworthy the prediction.

Mahalanobis Distance p-value: The p-value gives the fraction of training data with an MD greater than or equal to the one for the given sample, assuming normally distributed data. The smaller the p-value, the less trustworthy the prediciton. For highly non-normal X properties (e.g., fingerprints), the MD p-value is wildly inaccurate.

Structural Similar Compounds

Name	GLYBURIDE	GLIPIZIDE	C.I.PIGMENT RED 23
Structure	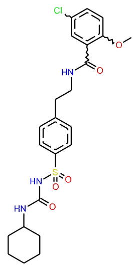	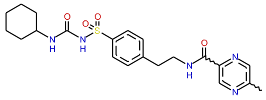	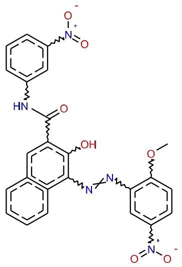
Actual Endpoint (-log C)	4.21661	3.94991	2.28997
Predicted Endpoint (-log C)	4.21035	3.95594	3.52921
Distance	0.787	0.792	0.825
Reference	UPJ-26452	NDA-17583	NTP 411 146

Model Applicability

Unknown features are fingerprint features in the query molecule, but not found or appearing too infrequently in the training set.

1. All properties and OPS components are within expected ranges.
2. Unknown ECFP_6 feature: 1997021792: [*]:[cH]:[cH]:[cH]:[*]
3. Unknown ECFP_6 feature: 1996163143: [*]:[cH]:[cH]:n:[*]
4. Unknown ECFP_6 feature: -677055651: [*]:[cH]:n:[cH]:[*]
5. Unknown ECFP_6 feature: -709633021: [*][c](:[*]):[cH]:n:[*]
6. Unknown ECFP_6 feature: -175146122: [*]C(=[*])[c](:[cH]:[*]):[cH]:[*]
7. Unknown ECFP_6 feature: 1430169877: [*]NC(=O)[c](:[*]):[*]
8. Unknown ECFP_6 feature: -177077903: [*]N[c](:[cH]:[*]):[cH]:[*]
9. Unknown ECFP_6 feature: 128986386: [*]\N=C(/C)\[c](:[*]):[*]
10. Unknown ECFP_6 feature: 560380707: [*]NN=C([*])[*]
11. Unknown ECFP_6 feature: 544048674: [*]C(=[*])NN=[*]

Feature Contribution

Top features for positive contribution			
Fingerprint	Bit/Smiles	Feature Structure	Score

FCFP_6	3	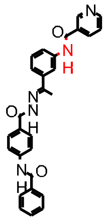<br>[*]N[*]	0.0924
FCFP_6	-2093839777	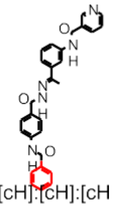<br>[*][c]1:[cH]:[cH]:[cH<br>]:[cH]:[cH]:1	0.078
ECFP_6	2099970318	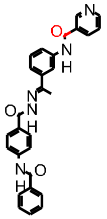<br>[*]C(=O)[*]	0.0766
**Top Features for negative contribution**			
**Fingerprint**	**Bit/Smiles**	**Feature Structure**	**Score**
FCFP_6	991735244	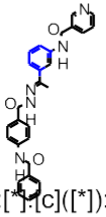<br>[*][c]1:[*]:[c]([*]):<br>[cH]:[cH]:[cH]:1	-0.134
ECFP_6	1564392544	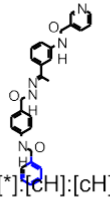<br>[*][c]1:[*]:[cH]:[cH]<br>:[cH]:[cH]:1	-0.133

FCFP_6	1	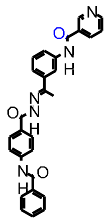 [*]=O	-

Sorafenib

TOPKAT_Chronic_LOAEL

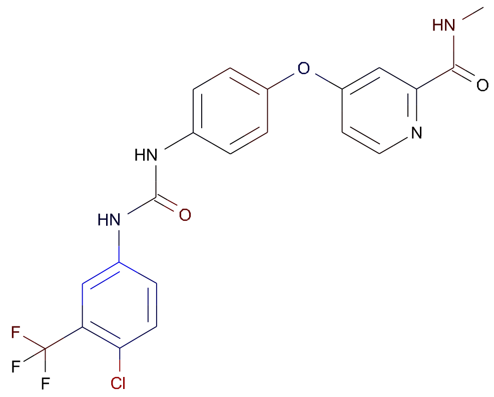

$C_{21}H_{16}ClF_3N_4O_3$
Molecular Weight: 464.82494

ALogP: 4.175

Rotatable Bonds: 6

Acceptors: 4

Donors: 3

Structural Similar Compounds

Name	GLYBURIDE	D & C RED 9	SODIUM ACIFLUORFEN
Structure	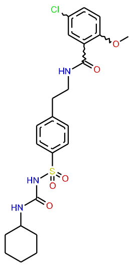	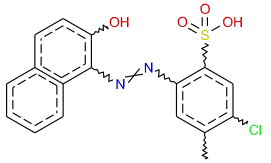	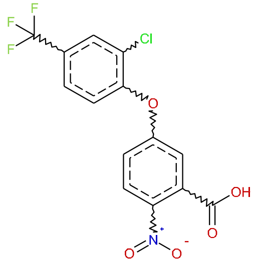
Actual Endpoint (-log C)	4.21661	3.87715	4.16036
Predicted Endpoint (-log C)	4.21035	3.6546	4.65915
Distance	0.636	0.722	0.736
Reference	UPJ-26452	NTP REPORT # 225	EPA COVER SHEET 0192;891101;(1)

Model Applicability

Unknown features are fingerprint features in the query molecule, but not found or appearing too infrequently in the training set.

1. All properties and OPS components are within expected ranges.
2. Unknown ECFP_6 feature: -1046436026: [*]F
3. Unknown ECFP_6 feature: 99947387: [*]:[c](:[*])Cl
4. Unknown ECFP_6 feature: 226796801: [*]C([*])([*])F
5. Unknown ECFP_6 feature: 1305253718: [*]:[c](:[*])O[c](:[*]):[*]
6. Unknown ECFP_6 feature: -677309799: [*][c](:[*]):n:[cH]:[*]
7. Unknown ECFP_6 feature: 1338334141: [*]C(=[*])NC
8. Unknown ECFP_6 feature: -177077903: [*]N[c](:[cH]:[*]):[cH]:[*]
9. Unknown ECFP_6 feature: 1336678434: [*][c](:[*]):[c](:[cH]:[*])C([*])([*])[*]
10. Unknown ECFP_6 feature: -649580166: [*]NC(=O)N[*]
11. Unknown ECFP_6 feature: -1952889961: [*]:[c](:[*])C(F)(F)F
12. Unknown ECFP_6 feature: 1413420509: [*]C(=[*])[c](:[cH]:[*]):n:[*]
13. Unknown ECFP_6 feature: 1996163143: [*]:[cH]:[cH]:n:[*]
14. Unknown ECFP_6 feature: 1430169877: [*]NC(=O)[c](:[*]):[*]
15. Unknown ECFP_6 feature: 864287155: [*]NC

Model Prediction

Prediction: 0.00483

Unit: g/kg_body_weight

Mahalanobis Distance: 30

Mahalanobis Distance p-value: 1.21e-024

Mahalanobis Distance: The Mahalanobis distance (MD) is a generalization of the Euclidean distance that accounts for correlations among the X properties. It is calculated as the distance to the center of the training data. The larger the MD, the less trustworthy the prediction.
Mahalanobis Distance p-value: The p-value gives the fraction of training data with an MD greater than or equal to the one for the given sample, assuming normally distributed data. The smaller the p-value, the less trustworthy the prediciton. For highly non-normal X properties (e.g., fingerprints), the MD p-value is wildly inaccurate.

Feature Contribution

Top features for positive contribution

Fingerprint	Bit/Smiles	Feature Structure	Score
ECFP_6	-176455838	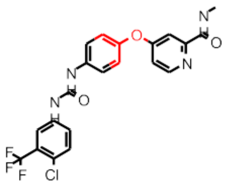 [*]O[c](:[cH]:[*]):[cH]:[*]	0.106
FCFP_6	32	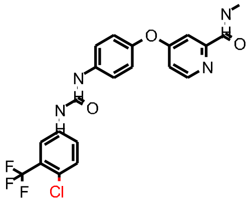 [*]Cl	0.101
FCFP_6	3	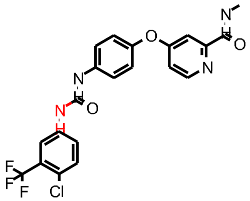 [*]N[*]	0.0924
**Top Features for negative contribution**			
**Fingerprint**	**Bit/Smiles**	**Feature Structure**	**Score**
FCFP_6	1	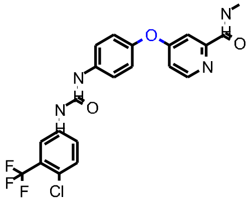 [*]=O	-0.102

ECFP_6	-1236483485	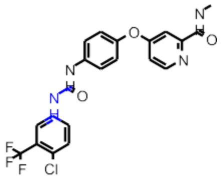 [*]C(=[*])N[c](:[*]):[*]	-0.0747
FCFP_6	203677720	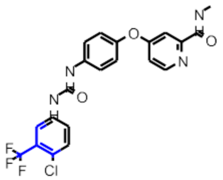 [*]C(=[*])[c](:[cH]:[*]):[cH]:[*]	-0.0713

Compound 10

TOPKAT_Rat_Maximum_Tolerated_Dose_Feed

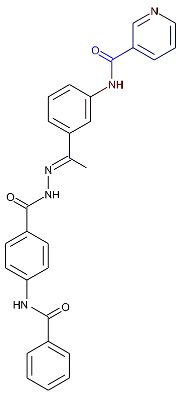

$C_{28}H_{23}N_5O_3$
Molecular Weight: 477.51391
ALogP: 3.082
Rotatable Bonds: 7
Acceptors: 5
Donors: 3

Model Prediction

Prediction: 0.117
Unit: g/kg_body_weight
Mahalanobis Distance: 9.42
Mahalanobis Distance p-value: 0.000352

Mahalanobis Distance: The Mahalanobis distance (MD) is a generalization of the Euclidean distance that accounts for correlations among the X properties. It is calculated as the distance to the center of the training data. The larger the MD, the less trustworthy the prediction.
Mahalanobis Distance p-value: The p-value gives the fraction of training data with an MD greater than or equal to the one for the given sample, assuming normally distributed data. The smaller the p-value, the less trustworthy the prediciton. For highly non-normal X properties (e.g., fingerprints), the MD p-value is wildly inaccurate.

Structural Similar Compounds

Name	SALICYLAZOSULFAPYRIDINE	FUROSEMIDE	C.I.PIGMENT RED 23
Structure	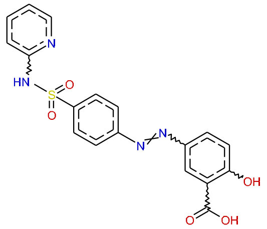	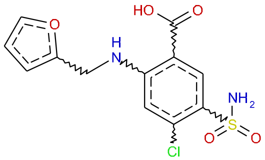	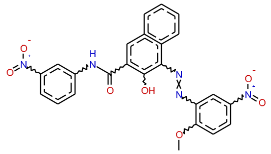
Actual Endpoint (-log C)	3.375	4.04236	2.30052
Predicted Endpoint (-log C)	2.80292	2.8614	3.55333
Distance	0.711	0.831	0.868
Reference	NCI/NTP TR-457	NCI/NTP TR-356	NCI/NTP TR-411

Model Applicability

Unknown features are fingerprint features in the query molecule, but not found or appearing too infrequently in the training set.

1. All properties and OPS components are within expected ranges.

Feature Contribution

Top features for positive contribution

Fingerprint	Bit/Smiles	Feature Structure	Score
FCFP_2	3	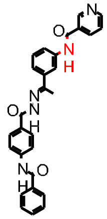 [*]N[*]	0.0737

FCFP_2	17	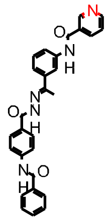 [*]:n:[*]	0.0441
FCFP_2	590925877	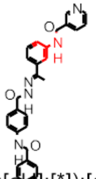 [*]N[c](:[cH]:[*]):[cH]:[*]	0.00762

Top Features for negative contribution

Fingerprint	Bit/Smiles	Feature Structure	Score
FCFP_2	1872154524	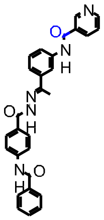 [*]C(=O)[*]	-0.105
FCFP_2	203677720	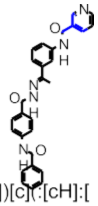 [*]C(=[*])[c](:[cH]:[*]):[cH]:[*]	-0.0829
FCFP_2	1	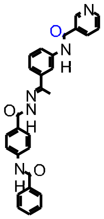 [*]=O	-0.0796

Sorafenib

TOPKAT_Rat_Maximum_Tolerated_Dose_Feed

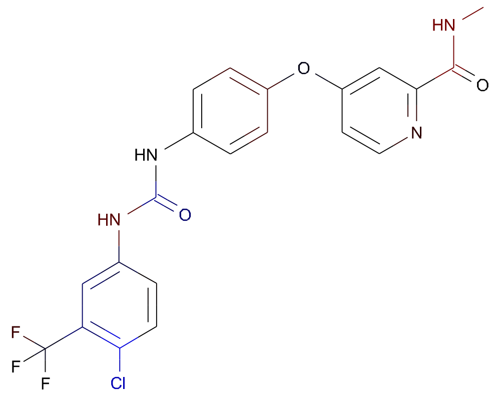

$C_{21}H_{16}ClF_3N_4O_3$
Molecular Weight: 464.82494

ALogP: 4.175

Rotatable Bonds: 6

Acceptors: 4

Donors: 3

Structural Similar Compounds

Name	FUROSEMIDE	PHENOLPHTHALEIN	DISPERSE YELLOW 3
Structure	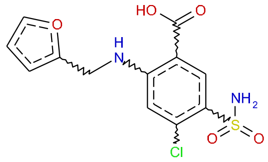	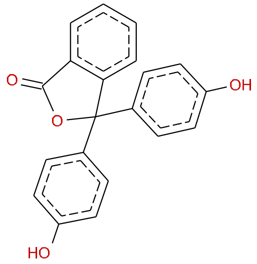	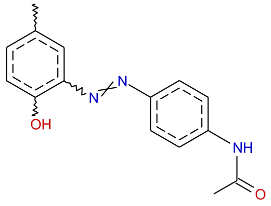
Actual Endpoint (-log C)	4.04236	2.20184	2.77703
Predicted Endpoint (-log C)	2.8614	2.8857	2.80195
Distance	0.741	0.780	0.799
Reference	NCI/NTP TR-356	NCI/NTP TR-465	NCI/NTP TR-222

Model Applicability

Unknown features are fingerprint features in the query molecule, but not found or appearing too infrequently in the training set.

1. All properties and OPS components are within expected ranges.

Feature Contribution

Top features for positive contribution

Fingerprint	Bit/Smiles	Feature Structure	Score
FCFP_2	-885550502	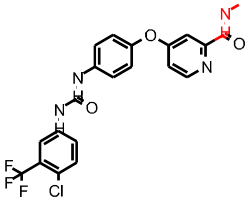 [*]C(=[*])NC	0.115

Model Prediction

Prediction: 0.0885

Unit: g/kg_body_weight

Mahalanobis Distance: 12.4

Mahalanobis Distance p-value: 1.76e-009

Mahalanobis Distance: The Mahalanobis distance (MD) is a generalization of the Euclidean distance that accounts for correlations among the X properties. It is calculated as the distance to the center of the training data. The larger the MD, the less trustworthy the prediction.
Mahalanobis Distance p-value: The p-value gives the fraction of training data with an MD greater than or equal to the one for the given sample, assuming normally distributed data. The smaller the p-value, the less trustworthy the prediciton. For highly non-normal X properties (e.g., fingerprints), the MD p-value is wildly inaccurate.

FCFP_2	3	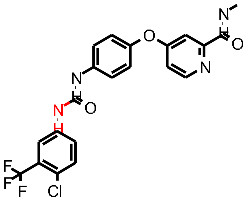 [*]N[*]	0.0737
FCFP_2	332760439	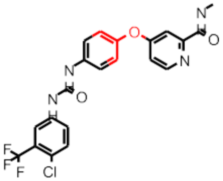 [*]O[c](:[cH]:[*]):[c H]:[*]	0.0611

Top Features for negative contribution

Fingerprint	Bit/Smiles	Feature Structure	Score
FCFP_2	71476542	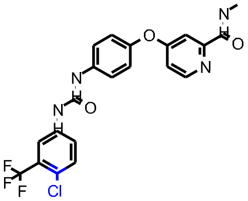 [*]:[c](:[*])Cl	-0.134
FCFP_2	1872154524	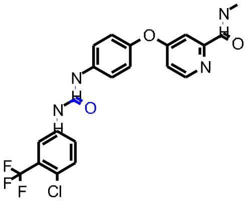 [*]C(=O)[*]	-0.105
FCFP_2	203677720	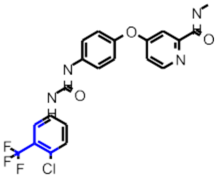 [*]C(=[*])[c](:[cH]:[*]):[cH]:[*]	-0.0829

Compound 10

TOPKAT_Rat_Maximum_Tolerated_Dose_Gavage

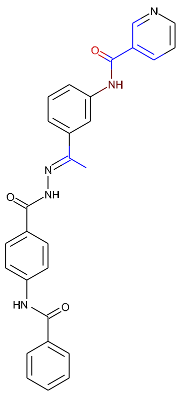

$C_{28}H_{23}N_5O_3$

Molecular Weight: 477.51391

ALogP: 3.082

Rotatable Bonds: 7

Acceptors: 5

Donors: 3

Structural Similar Compounds

Name	OCHRATOXIN	SULFISOOXAZOLE	PENICILLIN VK
Structure	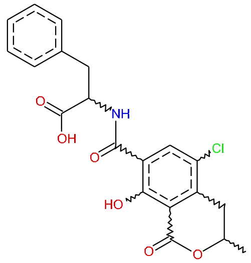	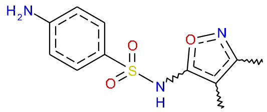	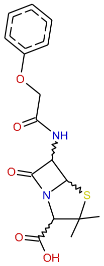
Actual Endpoint (-log C)	6.28396	2.82494	2.54455
Predicted Endpoint (-log C)	5.12358	3.0705	3.9702
Distance	0.921	1.081	1.282
Reference	NCI/NTP TR-358	NCI/NTP TR-138	NCI/NTP TR-336

Model Prediction

Prediction: 0.148

Unit: g/kg_body_weight

Mahalanobis Distance: 12.3

Mahalanobis Distance p-value: 3.08e-009

Mahalanobis Distance: The Mahalanobis distance (MD) is a generalization of the Euclidean distance that accounts for correlations among the X properties. It is calculated as the distance to the center of the training data. The larger the MD, the less trustworthy the prediction.

Mahalanobis Distance p-value: The p-value gives the fraction of training data with an MD greater than or equal to the one for the given sample, assuming normally distributed data. The smaller the p-value, the less trustworthy the prediciton. For highly non-normal X properties (e.g., fingerprints), the MD p-value is wildly inaccurate.

Model Applicability

Unknown features are fingerprint features in the query molecule, but not found or appearing too infrequently in the training set.

1. Molecular_Weight out of range. Value: 477.51. Training min, max, mean, SD: 68.074, 434.63, 171.13, 85.06.
2. Num_AromaticRings out of range. Value: 4. Training min, max, mean, SD: 0, 2, 0.5625, 0.693.
3. OPS PC6 out of range. Value: -2.6475. Training min, max, SD, explained variance: -2.4321, 2.9885, 1.256, 0.0488.
4. Unknown FCFP_2 feature: -1549192822: [*]\N=C(/C)\[c](:[*]):[*]
5. Unknown FCFP_2 feature: 581019816: [*]NN=C([*])[*]
6. Unknown FCFP_2 feature: -885520711: [*]C(=[*])NN=[*]

Feature Contribution

Top features for positive contribution

Fingerprint	Bit/Smiles	Feature Structure	Score

FCFP_2	1	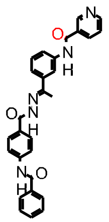 [*]=O	0.511
FCFP_2	3	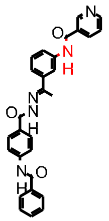 [*]N[*]	0.104

Top Features for negative contribution

Fingerprint	Bit/Smiles	Feature Structure	Score
FCFP_2	136597326	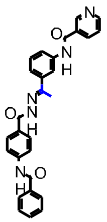 [*]C(=[*])C	-0.489
FCFP_2	203677720	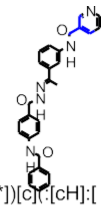 [*]C(=[*])[c](:[cH]:[*]):[cH]:[*]	-0.406
FCFP_2	1872154524	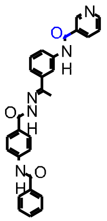 [*]C(=O)[*]	-0.307

Sorafenib

TOPKAT_Rat_Maximum_Tolerated_Dose_Gavage

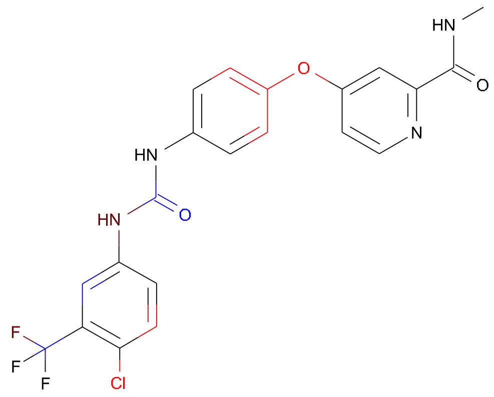

$C_{21}H_{16}ClF_3N_4O_3$
Molecular Weight: 464.82494
ALogP: 4.175
Rotatable Bonds: 6
Acceptors: 4
Donors: 3

Model Prediction

Prediction: 0.000918

Unit: g/kg_body_weight

Mahalanobis Distance: 12.2

Mahalanobis Distance p-value: 4.69e-009

Mahalanobis Distance: The Mahalanobis distance (MD) is a generalization of the Euclidean distance that accounts for correlations among the X properties. It is calculated as the distance to the center of the training data. The larger the MD, the less trustworthy the prediction.
Mahalanobis Distance p-value: The p-value gives the fraction of training data with an MD greater than or equal to the one for the given sample, assuming normally distributed data. The smaller the p-value, the less trustworthy the prediciton. For highly non-normal X properties (e.g., fingerprints), the MD p-value is wildly inaccurate.

Structural Similar Compounds

Name	OCHRATOXIN	SULFISOOXAZOLE	PENICILLIN VK
Structure	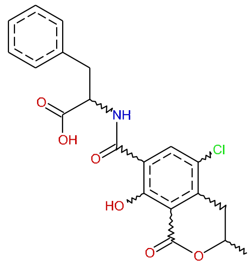	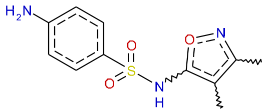	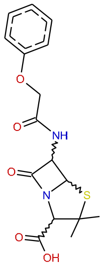
Actual Endpoint (-log C)	6.28396	2.82494	2.54455
Predicted Endpoint (-log C)	5.12358	3.0705	3.9702
Distance	0.758	0.997	1.159
Reference	NCI/NTP TR-358	NCI/NTP TR-138	NCI/NTP TR-336

Model Applicability

Unknown features are fingerprint features in the query molecule, but not found or appearing too infrequently in the training set.

1. Molecular_Weight out of range. Value: 464.82. Training min, max, mean, SD: 68.074, 434.63, 171.13, 85.06.
2. Num_AromaticRings out of range. Value: 3. Training min, max, mean, SD: 0, 2, 0.5625, 0.693.
3. OPS PC5 out of range. Value: -3.5737. Training min, max, SD, explained variance: -3.4, 4.1587, 1.489, 0.0686.
4. OPS PC7 out of range. Value: -3.8342. Training min, max, SD, explained variance: -2.8003, 2.9332, 1.16, 0.0416.
5. Unknown FCFP_2 feature: 1499521844: [*]NC(=O)N[*]
6. Unknown FCFP_2 feature: -1029533685: [*]:[c](:[*])C(F)(F)F
7. Unknown FCFP_2 feature: 136686699: [*]NC

Feature Contribution

Top features for positive contribution

Fingerprint	Bit/Smiles	Feature Structure	Score

FCFP_2	332760439	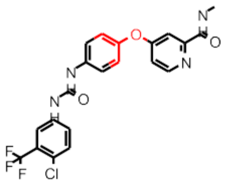 [*]O[c](:[cH]:[*]):[cH]:[*]	0.672
FCFP_2	32	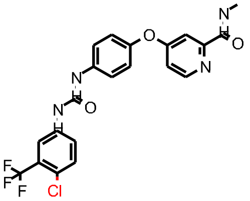 [*]Cl	0.526
FCFP_2	1	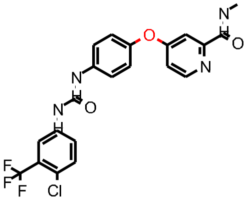 [*]=O	0.511
**Top Features for negative contribution**			
**Fingerprint**	**Bit/Smiles**	**Feature Structure**	**Score**
FCFP_2	203677720	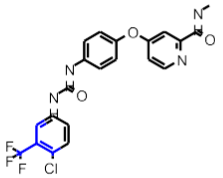 [*]C(=[*])[c](:[cH]:[*]):[cH]:[*]	-0.406
FCFP_2	1872154524	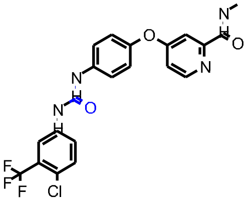 [*]C(=O)[*]	-0.307

FCFP_2	0	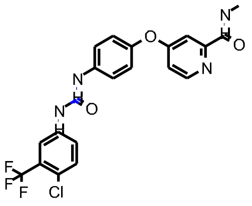 [*]C(=[*])[*]	-0.29

Compound 10

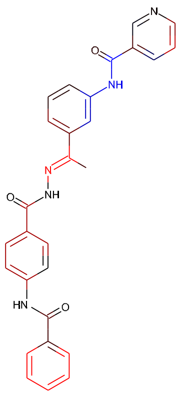

$C_{28}H_{23}N_5O_3$

Molecular Weight: 477.51391

ALogP: 3.082

Rotatable Bonds: 7

Acceptors: 5

Donors: 3

Structural Similar Compounds

Name	BENZENESULFONIC ACID; 2;2'-(4;4'-BIPHENYLYLENEDIVINYLENE)DI-; DISODIUM SALT (Na STRIPPED)	bis-OXATIN ACETATE	CARBAMIC ACID; N-(5-BENZOYLBENZIMIDAZOL-2-YL)-; METHYL ESTER
Structure	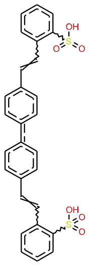	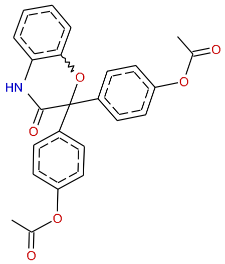	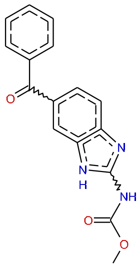
Actual Endpoint (-log C)	1.968	1.717	2.617
Predicted Endpoint (-log C)	1.72109	2.40947	2.2368
Distance	0.836	0.854	0.864
Reference	MVCRB3 2;193;73	NIIRDN 6;609;82	IYKEDH 19;735;88

Model Prediction

Prediction: 2.64

Unit: g/kg_body_weight

Mahalanobis Distance: 22.1

Mahalanobis Distance p-value: 1.54e-016

Mahalanobis Distance: The Mahalanobis distance (MD) is a generalization of the Euclidean distance that accounts for correlations among the X properties. It is calculated as the distance to the center of the training data. The larger the MD, the less trustworthy the prediction.
Mahalanobis Distance p-value: The p-value gives the fraction of training data with an MD greater than or equal to the one for the given sample, assuming normally distributed data. The smaller the p-value, the less trustworthy the prediciton. For highly non-normal X properties (e.g., fingerprints), the MD p-value is wildly inaccurate.

Model Applicability

Unknown features are fingerprint features in the query molecule, but not found or appearing too infrequently in the training set.

1. All properties and OPS components are within expected ranges.
2. Unknown ECFP_2 feature: 128986386: [*]\N=C(/C)\[c](:[*]):[*]
3. Unknown FCFP_6 feature: 16: [*]:[cH]:[*]
4. Unknown FCFP_6 feature: 1618154665: [*]:[cH]:[cH]:[cH]:[*]
5. Unknown FCFP_6 feature: 1747237384: [*]:[cH]:n:[cH]:[*]
6. Unknown FCFP_6 feature: 581019816: [*]NN=C([*])[*]
7. Unknown FCFP_6 feature: -885520711: [*]C(=[*])NN=[*]

Feature Contribution

Top features for positive contribution			
Fingerprint	Bit/Smiles	Feature Structure	Score

ECFP_6	642810091	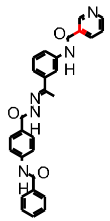 [*][c](:[*]):[*]	0.281
ECFP_6	-1897341097	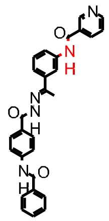 [*]N[*]	0.216
ECFP_6	1571214559	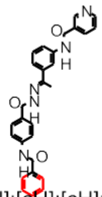 [*]1:[cH]:[cH]:[cH]:[cH]:[cH]:1	0.19
**Top Features for negative contribution**			
**Fingerprint**	**Bit/Smiles**	**Feature Structure**	**Score**
ECFP_6	655739385	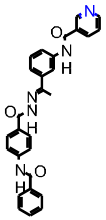 [*]:n:[*]	-0.239
ECFP_6	734603939	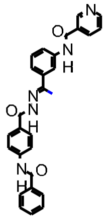 [*]C	-0.201

FCFP_6	-1549103449	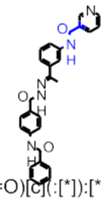 [*]NC(=O)[c](:[*]):[*]]	-

Sorafenib

TOPKAT_Rat_Oral_LD50

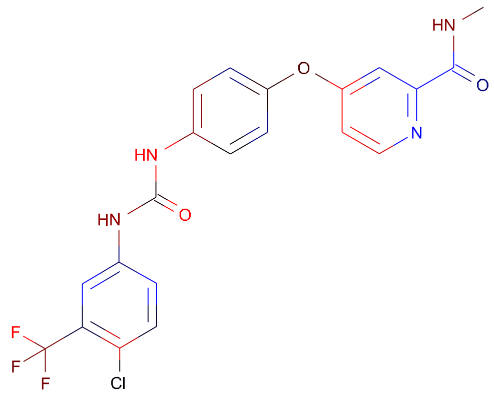

$C_{21}H_{16}ClF_3N_4O_3$
Molecular Weight: 464.82494
ALogP: 4.175
Rotatable Bonds: 6
Acceptors: 4
Donors: 3

Structural Similar Compounds

Name	FLUBENDAZOLE	PHOSPHORAMIDOTHIOIC ACID; ACETIMIDOYL-; O;O-bis-(p-CHLOROPHENYL)ESTER	BEZAFIBRATE
Structure	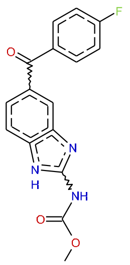	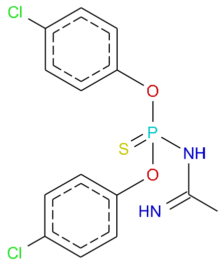	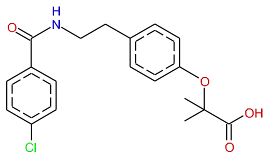
Actual Endpoint (-log C)	2.088	5.006	1.946
Predicted Endpoint (-log C)	2.69288	3.23989	2.54395
Distance	0.697	0.703	0.721
Reference	YRTMA6 9;11;78	FMCHA2 -;C149;89	ARZNAD 30;2023;80

Model Prediction

Prediction: 0.823
Unit: g/kg_body_weight
Mahalanobis Distance: 21
Mahalanobis Distance p-value: 1.93e-012

Mahalanobis Distance: The Mahalanobis distance (MD) is a generalization of the Euclidean distance that accounts for correlations among the X properties. It is calculated as the distance to the center of the training data. The larger the MD, the less trustworthy the prediction.
Mahalanobis Distance p-value: The p-value gives the fraction of training data with an MD greater than or equal to the one for the given sample, assuming normally distributed data. The smaller the p-value, the less trustworthy the prediciton. For highly non-normal X properties (e.g., fingerprints), the MD p-value is wildly inaccurate.

Model Applicability

Unknown features are fingerprint features in the query molecule, but not found or appearing too infrequently in the training set.

1. All properties and OPS components are within expected ranges.
2. Unknown FCFP_6 feature: 16: [*]:[cH]:[*]
3. Unknown FCFP_6 feature: 71476542: [*]:[c](:[*])Cl
4. Unknown FCFP_6 feature: 1747237384: [*]:[cH]:n:[cH]:[*]
5. Unknown FCFP_6 feature: 1618154665: [*]:[cH]:[cH]:[cH]:[*]
6. Unknown FCFP_6 feature: 136686699: [*]NC

Feature Contribution

Top features for positive contribution

Fingerprint	Bit/Smiles	Feature Structure	Score

FCFP_6	71953198	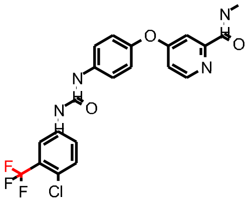 [*]C([*])([*])F	0.392
ECFP_6	-1046436026	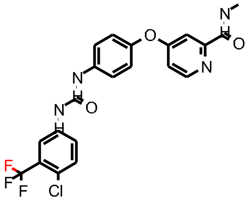 [*]F	0.349
ECFP_6	642810091	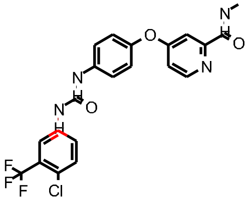 [*][c](:[*]):[*]	0.281
**Top Features for negative contribution**			
**Fingerprint**	**Bit/Smiles**	**Feature Structure**	**Score**
ECFP_6	226796801	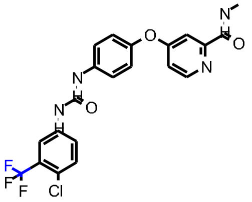 [*]C([*])([*])F	-0.32
ECFP_6	-817402818	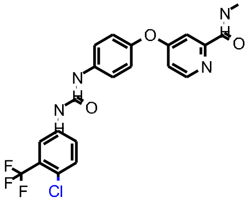 [*]Cl	-0.263

ECFP_6	-176455838	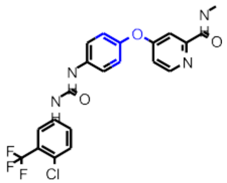 [*]O[c](:[cH]:[*]):[cH]:[*]	-
